# Identification and reproducibility of diagnostic DNA markers for tuber starch and yield optimization in a novel association mapping population of potato (*Solanum tuberosum* L.)

**DOI:** 10.1007/s00122-016-2665-7

**Published:** 2016-01-29

**Authors:** E. M. Schönhals, F. Ortega, L. Barandalla, A. Aragones, J. I. Ruiz de Galarreta, J.-C. Liao, R. Sanetomo, B. Walkemeier, E. Tacke, E. Ritter, C. Gebhardt

**Affiliations:** Max Planck Institute for Plant Breeding Research, Cologne, Germany; APPACALE S.A., Burgos, Spain; NEIKER, Vitoria-Gasteiz, Spain; Potato Germplasm Enhancement Laboratory, Obihiro University of Agriculture and Veterinary Medicine, Obihiro, Japan; Bioplant, Ebstorf, Germany

## Abstract

****Key message**:**

**SNPs in candidate genes*Pain*-*1,InvCD141*(invertases),*SSIV*(starch synthase),*StCDF1*(transcription factor),*LapN*(leucine aminopeptidase), and cytoplasm type are associated with potato tuber yield, starch content and/or starch yield.**

**Abstract:**

Tuber yield (TY), starch content (TSC), and starch yield (TSY) are complex characters of high importance for the potato crop in general and for industrial starch production in particular. DNA markers associated with superior alleles of genes that control the natural variation of TY, TSC, and TSY could increase precision and speed of breeding new cultivars optimized for potato starch production. Diagnostic DNA markers are identified by association mapping in populations of tetraploid potato varieties and advanced breeding clones. A novel association mapping population of 282 genotypes including varieties, breeding clones and Andean landraces was assembled and field evaluated in Northern Spain for TY, TSC, TSY, tuber number (TN) and tuber weight (TW). The landraces had lower mean values of TY, TW, TN, and TSY. The population was genotyped for 183 microsatellite alleles, 221 single nucleotide polymorphisms (SNPs) in fourteen candidate genes and eight known diagnostic markers for TSC and TSY. Association test statistics including kinship and population structure reproduced five known marker–trait associations of candidate genes and discovered new ones, particularly for tuber yield and starch yield. The inclusion of landraces increased the number of detected marker–trait associations. Integration of the present association mapping results with previous QTL linkage mapping studies for TY, TSC, TSY, TW, TN, and tuberization revealed some hot spots of QTL for these traits in the potato genome. The genomic positions of markers linked or associated with QTL for complex tuber traits suggest high multiplicity and genome wide distribution of the underlying genes.

**Electronic supplementary material:**

The online version of this article (doi:10.1007/s00122-016-2665-7) contains supplementary material, which is available to authorized users.

## Introduction

Potatoes are, besides being one of the world’s most important food crops, a renewable resource of the starch biopolymer (Zobel 1988), which has a wide range of industrial applications. The major industrial sectors for potato starches are the food, paper, general and textile industries (Ellis et al. 1998). Starch constitutes between 11 and 45 % of the tuber fresh weight (Jansen et al. 2001). The tuber starch content of middle European varieties ranges from 10 to 17 % for table potatoes, from 14 to 20 % for processing potatoes (e.g., chips, French fries) and reaches up to 25 % in industrial potatoes (personal communication H.-R. Hofferbert, Böhm-Nordkartoffel Agrarproduktion, Ebstorf, Germany). The variability of tuber starch content (TSC) is caused by natural DNA variation at multiple genetic loci and by environmental factors. One of the most relevant traits for industrial starch production is starch yield, the amount of starch that is obtained per unit of arable land. Hence, one objective in breeding cultivars for industrial starch production is the maximization of tuber starch yield (TSY). This is not simply achieved by maximizing tuber yield because tuber yield (TY), that is the total tuber weight per plant, per plot or per unit of arable land, is negatively correlated with tuber starch content (Li et al. 2013; Urbany et al. 2011). To complicate matters further, higher tuber yield and starch content are both correlated with later plant maturity (Urbany et al. 2011; van Eck 2007), which is an undesirable agronomic character in potato cultivation. Yield is probably the most complex polygenic trait and is strongly influenced by environmental conditions and agricultural practices, whereas plant maturity is a relatively stable character. The assessment of tuber yield in breeding programs requires multi-year and location trials and is not reliable in the early years of selection due to lack of sufficient tuber numbers. The early diagnosis of tuber starch content, yield and starch yield potential in potato breeding populations by means of DNA markers could facilitate the combination of superior alleles for high starch yield in novel cultivars.

Diagnostic DNA markers are either derived from DNA variation in loci that directly contribute to the heritable variation of complex traits, or are in strong linkage disequilibrium (LD) with such loci. The identification of diagnostic DNA markers for complex agronomic traits such as TSC, TY, and TSY requires association mapping in populations of breeding materials. The concept of association mapping has been adopted from human population genetics (Stranger et al. 2011) and takes advantage of historical recombination events in populations of individuals related by descent. In crops, association mapping populations are assembled from genetic resources that ideally represent the genetic and phenotypic diversity of the species. Such populations are phenotyped for agronomic traits of interest and genotyped with DNA markers either at particular candidate loci or with genome wide distribution. The markers are then tested for association with the traits. Marker–trait associations are observed when a specific marker allele is co-inherited with a specific trait allele over a number of meiotic generations due to identity between marker and trait locus or close physical linkage between them. Marker–trait association can also occur between unlinked loci due to population substructure, which in crops can result from directed selection for specific trait complexes. Association test statistics have to take into account the possibility of false positive association due to population substructure (Flint-Garcia et al. 2003; Zhu et al. 2008). Association mapping of starch and yield related traits has been performed over the last 10 years in major cereal crops such as maize, rice, wheat, barley and sorghum (Borba et al. 2010; Cook et al. 2012; Matthies et al. 2014; Pauli et al. 2014; Reif et al. 2011; Sukumaran et al. 2012; Wilson et al. 2004; Xu et al. 2013) and in the major tuber crop potato (D’hoop et al. 2014; Li et al. 2005, 2008, 2010; Schreiber et al. 2014; Urbany et al. 2011).

The biochemical and genetic basis of starch biosynthesis and breakdown is well known, thanks to numerous molecular studies in model and crop plants including potato (Hofius and Börnke 2007; Tetlow et al. 2004; Zeeman et al. 2010). This together with linkage mapping of quantitative trait loci (QTL) for TSC and TY in experimental populations (Schäfer-Pregl et al. 1998) and of genes functional in plant carbohydrate metabolism and transport (Chen et al. 2001) provided the foundation for a candidate gene approach towards the discovery of diagnostic markers for TSC and TSY in potato and eventually the genes that explain the natural variation of these traits. Association mapping of TSC, TY and TSY has been performed previously in a population of 220–240 tetraploid potato genotypes from three commercial breeding programs in Germany (referred to as CHIPS-ALL population). DNA polymorphisms in genes known to function in plant carbohydrate metabolism and co-localizing with QTL for TSC and/or TY were used for genotyping. This identified a first series of DNA markers which were associated with increased or decreased TSC and TSY (Draffehn et al. 2010; Li et al. 2008, 2010, 2013; Schreiber et al. 2014). Very few marker associations with tuber yield have been found so far, probably because most of the tested candidate genes were chosen primarily for their function in carbohydrate metabolism and transport (Li et al. 2008) or in enzymatic discoloration of tubers upon mechanical damage (tuber bruising) (Urbany et al. 2011) and less for their potential relevance for tuber yield. Alternatively, the effects of single alleles on yield might be too small to be detected with confidence in populations of 200–250 individuals.

Marker–trait associations discovered in a given association panel may or may not be reproducible in other populations of the same species. Reasons for failure to reproduce a marker–trait association are different recombination history, different allele frequencies, genotype by environment interactions, different sampling strategies and phenotyping standards. Similar to association studies in human populations (Stranger et al. 2011), marker–trait associations should be therefore reproduced in independent populations (D’hoop et al. 2014; Schreiber et al. 2014; Zhang et al. 2014) or, in the case of crop plants, by marker-assisted selection (Li et al. 2013). To this purpose we established a novel association mapping population of tetraploid potato (subsequently referred to as the QUEST population), which was evaluated in Northern Spain for tuber yield (TY), starch content (TSC), and number (TN). Approximately half of the population was also scored for plant maturity (PM). The QUality starches by Exploiting new breeding tools in *Solanum tuberosum* (QUEST) population was genotyped with markers that were previously shown to be associated with TSC, TSY and in one case with TY in the CHIPS-ALL population, which has been phenotyped in Northern Germany (Li et al. 2008). The diagnostic markers described so far explain only part of the variation of TSC and TSY and very little of TY. We evaluated therefore single nucleotide polymorphisms (SNPs) in five new candidate genes for association with the agronomic traits. The plastidial Calvin cycle protein CP12 is involved in the regulation of enzymes of the Calvin-Benson cycle (Graciet et al. 2004) and might influence tuber yield via modulating the flux of carbon from source to sink tissues. Its antisense suppression in tobacco had severe effects on carbon partitioning and growth (Howard et al. 2011). The starch synthase IV (*SSIV*) controls granule number and size of transient starch in Arabidopsis (Roldán et al. 2007). Plastidial phosphoglucoisomerase 1 (PGI1) partakes in the metabolic network that controls source-sink relationships by providing phosphate sugar metabolites for the transient accumulation of starch in photosynthetic leaves (Geigenberger 2011; Yu et al. 2000), which could be relevant for tuber starch content and yield. *StCDF1* is a cycling DOF (DNA binding with One Finger) transcription factor that was recently shown to control day length dependent tuberization, which is related to plant maturity. *StCDF1* co-localizes with a major QTL for plant maturity on potato chromosome V (Collins et al. 1999; Kloosterman et al. 2013; Oberhagemann et al. 1999). Due to the correlation of TSC and TY with plant maturity, natural variation at the *StCDF1* locus might have as well an effect on tuber yield and starch content. The transcription factor *StBEL5* is also a candidate gene for tuber yield as it plays a role in tuber formation and growth (Chen et al. 2003; Sharma et al. 2014).

Potato plastid and mitochondrial DNA also show DNA variation (Hosaka 1986; Lössl et al. 1999), which could be, in concert with nuclear genes, important for TSC, TY, and TSY. We therefore tested DNA markers diagnostic for different cytoplasm types (Hosaka and Sanetomo 2012) for association with TSC, TY, TSY, TN, and TW.

In this paper we report on (i) the properties of the new association mapping population QUEST, (ii) the reproducibility of diagnostic markers for tuber starch content and starch yield in this population, and (iii) novel associations of SNPs in candidate genes that were selected with emphasis on their potential role for yield related traits.

## Materials and methods

### Plant material and experimental design

An association mapping population of 282 potato genotypes was assembled, to which we refer as the QUEST population. It consisted of 191 tetraploid cultivars (CUL), 73 tetraploid breeding clones (BRE), and 18 landraces (LAN) (Online Resource 1). Two cultivars (cvs Ponto and Panda) were shared with the CHIPS-ALL association mapping population (Li et al. 2008). Two subpopulations were grown and evaluated at the breeding stations of APPACALE (Burgos/Spain, latitude 42.34′) (157 genotypes) and NEIKER-Tecnalia (Vitoria-Gasteiz/Spain, latitude 42.85′) (175 genotypes) in 2 years (2010 and 2011). The trials were planted between mid-April and the beginning of May and harvested at the end of September. Fifty standard cultivars were grown at both sites. The trials were planted in an Augmented Design (Petersen 1985) in blocks of 25 cultivars with three varieties (Desiree, Kennebec, and Jaerla) as testers in each block. The experimental setup slightly differed for each year at the two sites. At NEIKER, ten tubers per genotype were planted in one block in both years. In 2010 and 2011, four representative single plants were harvested in each plot and phenotyped individually. At APPACALE, two tubers per genotype were planted in 2010. Both plants were harvested and bulked for phenotypic analysis. In 2011, six plants per plot were grown and bulked for phenotypic analysis.

### Phenotypes

Tuber yield (TY, g/plant) and tuber number (TN) per plant were determined. Tuber weight (TW, g/tuber) was calculated by dividing TY by TN. Tuber starch content (TSC, percent fresh weight) was calculated from the specific gravity measured according to (Von Scheele et al. 1937). Tuber starch yield (TSY, g/plant) was calculated as the product of TSC by TY. One hundred and fifty-four genotypes grown at the APPACALE site were scored for plant maturity (PM_1_) using a 1–9 scale where 1 indicates very late and 9 very early plant maturity. In addition, scores from 1 (very late) to 9 (very early) for plant maturity (PM_2_) were extracted for 90 varieties from passport data in The European Cultivated Potato Database (http://www.europotato.org) and the ‘Beschreibende Sortenliste Kartoffeln’ of the German Bundessortenamt and the Austrian Federal Office for Food Safety (Online Resource 1).

### Phenotypic data analysis

All statistical analyses were performed with the statistical software R (R Core Team 2013) if not stated otherwise. The phenotypic data of the two sites in 2010 and 2011 were compiled and corrected for the experimental design, using a linear model with two factors, implemented in the R package ‘stats’ using the function *lm()*. Correction was solely performed for the phenotypic data of APPACALE, as there was not sufficient data to correct for the experimental design at NEIKER in both years. The factor *block* had 7 levels. The three levels of the factor *tester* corresponded to the three tester varieties that were planted in each block. First the correction factors were estimated and then the phenotypic data were corrected for the block effects.

Adjusted entry means over 2 years and two sites were calculated from corrected data (APPACALE) and primary data (NEIKER) according to Li et al. (2008) using the linear model:

$$y \, = \, \mu \, + {\text{ genotype }} + {\text{environment }} + \, \varepsilon.$$

The set of 50 cultivars grown at both sites in both years formed the basis for the adjustment. The levels of the factor *genotype* corresponded to the number of genotypes in the trial and the four levels of the factor *environment* were: APPACALE trial in 2010, APPACALE trial in 2011, NEIKER trial in 2010 and NEIKER trial in 2011. When applied to one generation with no selection, the heritability (*H*^*2*^) is an indicator for the repeatability of the phenotypic data. It was calculated from the ratio between genotypic and phenotypic variance. Variance components were estimated by a mixed linear model with *environment* as fixed and *genotype* as random term:

$${\text{y }} = \, \mu \, + {\text{genotype}} + {\text{environment }} + \, \varepsilon.$$

The mixed linear model was implemented in the R package ‘lme4′ by the function *lmer()*. Based on the estimated variance components, the heritability was calculated according to

$$H^{2} = \sigma^{2}_{\text{g}} / \, \left[ {\sigma^{2}_{\text{g}} + \, \left( {\sigma^{2}_{\text{e}} /n} \right)} \right]$$

where $\sigma^{2}_{\text{g}}$ represents the variance component for the genotypic main effect, $\sigma^{2}_{\text{e}}$ represents the variance component for the residuals and *n* the number of environments.

Correlations between phenotypic traits were calculated based on the adjusted entry means for TSC, TY, TSY, TN, and TW, and simple arithmetic means for PM_1_ and PM_2_ using SPSS statistical software (IBM, Germany).

### DNA extraction

Total genomic DNA was extracted from 20 mg freeze dried leaves of each genotype, using the DNeasy Plant Mini Kit (Qiagen, Hilden, Germany) according to the manufacturer’s protocol. An additional washing step with 500 μl 96 % ethanol was performed prior to the elution of DNA from the column. DNA concentration and quality were assessed using the Qubit dsDNA BR Assay Kit (Invitrogen, Karlsruhe, Germany) and standard electrophoresis on 1 % agarose gels.

### Amplicon sequencing of candidate genes and SNP scoring

SNP markers were newly developed for the candidate genes *CP12*-*2* (Calvin cycle protein 2), *SSIV* (starch synthase IV), *PGI1*-*4* (phosphoglucoisomerase 1 on chromosome IV), *StCDF1* (cycling DOF factor 1), and *StBEL5* (BEL family transcription factor) and scored in the QUEST population. Sequence information was retrieved with accession numbers from the NCBI database (NCBI; http://www.ncbi.nlm.nih.gov/). The sequences were BLASTed against the potato genome sequence (version v4.03) (PGSC 2011; Sharma et al. 2013) to obtain loci, transcript or super scaffold numbers using the PGSC Genome Browser (http://solanaceae.plantbiology.msu.edu/cgi-bin/gbrowse/potato/). The position on the pseudomolecule, the genomic sequence as well as the exon–intron structure of the PGSC representative gene model was retrieved. Gene-specific primers were designed to be located at the borders of exons. Genes and primers used for amplicon sequencing are shown in Table 1. The standard PCR reaction was performed in 25 µl reaction volume, containing 50 ng genomic DNA, 10 mM Tris–HCl pH 8.3, 50 mM KCl, 1.5 mM MgCl_2_, 0.1 % Triton X-100, 100 µM of each dNTP (Roth, Karlsruhe, Germany), 0.4 µM of each primer, 1U Taq Polymerase (Ampliqon, Odense M, Denmark), and deionized water (Merck KGaA, Darmstadt, Germany). PCR conditions were: 3 min initial denaturation at 95 °C, then 35 cycles of denaturation 20 s at 94 °C, annealing 40 s at *T*_a_ (Table 1) and elongation 30 s per 500 bp at 72 °C, followed by 10 min final elongation at 72 °C. The amplification result was checked on 1.5 % agarose gels. PCR products were purified with Illustra ExoStar (GE Healthcare Europe GmbH, München, Germany) for 15 min at 37 °C, followed by 15 min at 80 °C. PCR fragments were custom sequenced by the Max Planck-Genome-Centre Cologne (http://mpgc.mpipz.mpg.de/home/) using the dideoxy chain-termination sequencing method, ABI PRISM Dye Terminator Cycle Sequencing Ready Reaction Kit and Applied Biosystems (Weiterstadt, Germany) 3730XL Genetic Analyzer sequencer.

**Table 1:** PCR information for five new candidate loci and the specific allele *InvCD141*-*Sa* evaluated for association with tuber traits in the QUEST population

Locus	Chromosome: position (bp) (pseudomolecule version v4.03)	Locus ID PGSC0003…	Primer sequence (5′–3′)	*T*_a_ (°C)	Amplicon size (bp)	Scored SNPs/indels
*CP12*-*2*	chr01:62723718..62724360	DMG400009042	F_GGCAACAATTGCTGGTGTTA^a^ R_GCCTAATTCATAGCATTCAAGATTC	59	453	11/1
*SSIV*	chr02:30142740..30152314	DMG400008322	F_CTCAATGAAGCTCGTGTCCA R_CAAAATTCCGAAGGCATCTC^a^	50	869	15/0
*PGI1*-*4*	chr04:64775429..64782339	DMG400012910	F_AGCATCTACTCACCTTCTTCATCTTTC R_TGCAAACTGGCAAACAGCTT^a^	56	492	14/1
*StCDF1*	chr05:04538880..04541736	DMG400018408	F_CCGCGATGTAATAGCATGGA^a^ R_GTTCCAAGGGTTTGCTACGG	56	591	16/0
*StBEL5*	chr06:54709882..54713896	DMG400005930	F_CGATTATGGAAGCCAATGGT R_GGAAATCGCTTATTCCCACTC^a^	57	668	20/0
*InvCD141*-*Sa*	chr10:55851945..55856805	DMG402028252	F_GGGCAACATTATTTGGGCT R_TTGGCTTTTGTTGATGGTTTTA	65–60	295	–^b^

Primers, annealing temperature (T_a_), amplicon size and number of SNPs/indels scored in the amplicons are shown

^a^Primer used for amplicon sequencing

^b^Allele-specific assay

SNPs in nine candidate genes *AGPaseS* (ADP-glucose pyrophosphorylase S, chromosome I), *SssI* (soluble starch synthase I, chromosome III), *PGM1*-*3* (plastidial phosphoglucomutase, chromosome III), *PHO1b* (L-type starch phosphorylase b, chromosome V)*, BMY*-*8/2* (beta- amylase, chromosome VIII)*, INV*-*8/2* (invertase, chromosome VIII)*, PWD* (phosphoglucan water dikinase, chromosome IX)*, InvCD141* (apoplastic invertase, chromosome X), and *LapN* (leucine aminopeptidase N, chromosome XII) were analysed as described previously (Draffehn et al. 2010; Fischer et al. 2013; Schreiber et al. 2014). Amplicon sequences were aligned and SNPs were detected with NovoSNP software (Weckx et al. 2005). SNP allele dosage was scored using both the Data Acquisition & Data analysis software DA × 8.1 (Van Mierlo Software Consultancy) and manual scoring. The predominantly bi-allelic SNPs were coded into five genotype classes (0, 1, 2, 3, 4), with 0 being the class homozygous for the SNP allele corresponding to the potato genome sequence (AAAA), 1, 2, and 3 corresponding to the three heterozygous genotypes (AAAB = 1, AABB = 2, ABBB = 3) and 4 being the class homozygous for the alternative SNP allele (BBBB). SNPs with more than two alleles were excluded from further analysis.

### Allele-specific PCR markers

Allele-specific PCR assays for the candidate gene markers *Pain1*-*8c* (soluble acid invertase, chromosome III), *Stp23*-*8b(PHO1a*-*H*_*A*_*)* (L-type starch phosphorylase a, chromosome III), *StpL(PHO1b)*-*3b, StpL(PHO1b)*-*3e* (both L-type starch phosphorylase b, chromosome V), *GP171*-*a* (chromosome VIII), *InvGE*-*6f* (apoplastic invertase, chromosome IX), and *Rca*-*1a* (ribulose bisphosphate carboxylase/oxidase activase, chromosome X) were performed as described (Li et al. 2005, 2008, 2010; Schreiber et al. 2014). An allele-specific assay was newly developed for the *InvCD141*-*Sa* allele (Draffehn et al. 2010; Schreiber et al. 2014). *InvCD141*-*Sa* was amplified from 50 ng genomic DNA template in 15 µl total volume containing 10 mM Tris–HCl pH 8.3, 50 mM KCl, 1.5 mM MgCl_2_, 0.1 % Triton X-100, 0.2 mM of each dNTP, 1 μM of each primer (Table 1) and 1.5U Taq Polymerase (Ampliqon, Odense M, Denmark). PCR conditions were: Initial denaturation for 3 min at 94 °C; 5 cycles of touch down PCR: 60 s denaturation at 94 °C, 60 s annealing at 65 °C, decreasing T_a_ by 1 °C per cycle, 60 s at 72 °C; then 30 cycles with 45 s denaturation, 45 s annealing at 60 °C and 45 s at 72 °C, followed by a 5 min final extension step at 72 °C. Allele-specific markers were scored as absent (0) or present (1).

### Microsatellite markers

The QUEST population was genotyped for 183 alleles at 29 microsatellite loci (Online Resource 2) (Feingold et al. 2005; Frary et al. 2005; Ghislain et al. 2009; Milbourne et al. 1998; Odeny et al. 2010). The main selection criteria for the microsatellite markers were: the primers amplified a single locus in the genome, DNA fragments were clearly distinguishable on the gel and were polymorphic in the population. At least one marker per chromosome arm was selected. Microsatellite markers were amplified in 25 µl reaction volume, containing 50 ng genomic DNA, 8 mM Tris–HCl pH 8.3, 40 mM KCl, 6.4 mM MgCl_2_, 0.08 % Triton X-100, 160 µM of each dNTP (Roth, Karlsruhe, Germany), 0.2 µM of each primer, 1U Taq polymerase (Ampliqon, Odense M, Denmark) in deionized water (Merck KGaA, Darmstadt, Germany). PCR conditions were: 3 min denaturation at 94 °C, 2 min at *T*_a_ (Online Resource 2), 90 s at 72 °C, followed by 29 cycles denaturation 60 s at 94 °C, annealing 60 s at *T*_a_ and elongation 45 s at 72 °C, final elongation 5 min at 72 °C (Provan et al. 1996). For markers with *T*_a_ 60-54 °C, touch down PCR was performed with the same procedure, lowering the temperature by 1 °C per cycle until the final annealing temperature was reached. PCR results and band intensity was assessed on 2 % standard agarose gels. Microsatellite alleles were separated on Spreadex gels (Elchrom Scientic AG, Cham, Switzerland) in the Elchrom SEA 2000 system (Elchrom Scientific AG, Cham, Switzerland) according to the supplier’s instruction manual. Allele sizes were estimated in comparison to the M3 size standard marker (Elchrom Scientic AG, Cham, Switzerland). The microsatellite alleles were treated as dominant markers and scored as absent (0) or present (1).

### Cytoplasm type markers

The QUEST population was genotyped with cytoplasm-specific markers according to (Hosaka and Sanetomo 2012). Six cytoplasm types were distinguished: T type cytoplasm, most prevalent in *S. tuberosum* spp. *tuberosum*, D type cytoplasm from *S. demissum*, A type cytoplasm, most prevalent in *S. tuberosum* ssp. *andigena*, P type cytoplasm introduced from *S. phureja*, M type cytoplasm (mother type, or an ancestral type of Andean cultivated potatoes) and W type cytoplasm from unidentified wild species including the *S. stoloniferum* derived sub-type W/γ (Hosaka and Sanetomo 2012).

### Population structure and kinship analysis

Population structure and kinship were analysed based on 183 microsatellite alleles at 29 loci. Two approaches were applied for detecting population structure: principle coordinate analysis (PCoA) (Gower 1966) and a Bayesian clustering approach. PCoA was based on pairwise genetic distances between the genotypes that were calculated from the microsatellite marker data. Jaccard’s distances were calculated with the R package ‘prabclus’ using the function *jaccard()* under default settings. Distances were transformed by square root transformation to obtain euclidean properties, following (Reif et al. 2005). Principal coordinate analysis was performed based on Jaccard’s distances between cultivars with the R package ‘stats’ (*cmdscale()*). The custom-made script was provided by Benjamin Stich (Max-Planck Institute for Plant Breeding Research, Cologne, Germany). The explained variance of each principal coordinate was calculated. Population structure was further determined by analyzing the microsatellite marker data with the software STRUCTURE 2.3.4 (Pritchard et al. 2000). Burn-in time as well as iteration number was set to 100,000 with 10 repetitions, testing the probability of 20 subpopulations in the QUEST population. The results were submitted to STRUCTURE HARVESTER (Earl and vonHoldt 2012) and the most likely number of subpopulations K was determined according to (Evanno et al. 2005). The kinship matrix *K* was calculated using the R package EMMA (Kang et al. 2008).

### Association analysis

Markers with more than 5 % of missing values were excluded from the analysis. As the GAPIT software did not tolerate missing data, up to 5 % missing marker data were replaced at random, according to the proportion of genotypic classes within each individual marker. For SNP markers up to five classes (0, 1, 2, 3, 4) were replaced, while for microsatellite and indel markers two classes (0, 1) were replaced. The approach was tested by association analysis (see below) of a subset of markers, where missing values were replaced three times at random. The differences between the *p* values were negligible.

The two-step approach as described by (Stich et al. 2008) was chosen for the association analysis. First, adjusted entry means were calculated for TY, TN, TW, TSC, and TSY (see above), which were then used for association analysis using a mixed linear model, which accounted for population structure and kinship. The mixed linear model equation for the *PK* method (Stich et al. 2008; Yu et al. 2006) was:

$$y{\mkern 1mu} = {\mkern 1mu} \mu {\mkern 1mu} + {\mkern 1mu} P{\mkern 1mu} + \underset{\raise0.3em\hbox{$\smash{\scriptscriptstyle-}$}}{K} + {\mkern 1mu} \varepsilon.$$

Population structure was accounted for by the *P* matrix (fixed term), wherefore the first 11 principal coordinates were extracted from PCoA, which explained in total 10 % of the variance. *K* represented the kinship between genotypes (random term). The analysis was performed with a mixed linear model implemented in the software package ‘GAPIT’ (Lipka et al. 2012). A GAPIT script for the analysis of data of tetraploid species was kindly provided by Alexander E. Lipka (Department of Crop Sciences, University of Illinois, W-201B Turner Hall, 1102 S Goodwin Ave, Urbana IL 61801), which was modified to accept besides the two homozygous (coded 0 and 4) the three heterozygous genotype classes (coded 1, 2, and 3) of a bi-allelic SNP with genotypes *AAAA, AAAB, AABB, ABBB,* and *BBBB*. Only additive marker effects were included in the model. Associations of markers with less than 1 % frequency of the minor frequency allele (MFA) were reported but are considered unreliable. In addition to the mixed model, the data were analyzed with a simple general linear model (GLM): $y \, = \, \mu \, + {\text{ marker }} + \, \varepsilon .$

### Linkage disequilibrium (LD)

LD was estimated for all pairs of SNP markers with a Chi-square test, based on the allele frequencies at all SNP loci. The *p*-values were corrected for multiple testing with the Bonferroni-Holm correction. The analysis was performed using a custom-made R script provided by Benjamin Stich (MPI for Plant Breeding Research, Cologne, Germany).

## Results

### Phenotypic analysis

**Fig. 1:**
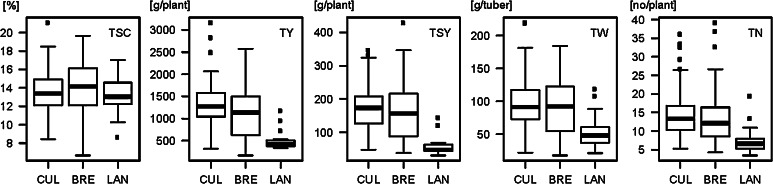
Boxplots of the adjusted entry means for tuber starch content (TSC), tuber yield (TY), tuber starch yield (TSY), tuber number (TN), and tuber weight (TW) evaluated in two-year field trials in 191 tetraploid cultivars (CUL), 73 tetraploid breeding clones (BRE) and 16 Andean landraces (LAN) of the QUEST population

The 282 tetraploid genotypes of the QUEST population including cultivars, breeding clones, and landraces were assessed for tuber traits at two trial sites in Northern Spain in years 2010 and 2011. Phenotypic data were obtained for 280 genotypes. Adjusted entry means were calculated for tuber yield (TY), number (TN), weight (TW), starch content (TSC), and starch yield (TSY) (Online Resource 1). Cultivars, breeding clones and landraces had similar mean TSC with higher variability among the breeding clones, whereas for TY, TN, TW, and TSY the landraces clearly showed lower mean values compared to cultivars and breeding clones (Fig. 1). Plant maturity was scored for 154 genotypes of the QUEST population grown at the APPACALE site (PM_1_). Additional maturity scores for 90 varieties (PM_2_) were derived from public variety descriptions (Online Resource 1).

The heritability (*H*^2^) or in this case the repeatability of the phenotypic analysis of tuber traits was between 0.73 and 0.83 for the complete population including the landraces (QUEST(+LAN), *n* = 280) and between 0.69 and 0.84 for the population without the landraces (QUEST(-LAN), *n* = 264). The highest *H*^2^ value was obtained for TSC followed by TSY, TY, TN, and TW (Table 2). All traits were correlated with each other, except TSC and TY, PM and TW, and TY and PM_2_, which were not correlated. Increased tuber starch content was correlated with higher tuber number, lower tuber weight and later plant maturity. Increased yield was correlated with higher tuber number and weight, and also with later plant maturity. Tuber number and weight were inversely correlated (Table 2).

**Table 2:** Bivariate (Pearson) correlation coefficients between traits, and heritabilities *H* ^2^

Trait	TSC	TY	TSY	TN	TW	PM_1_		*H* ^2^ [QUEST(+LAN)]	*H* ^2^ [QUEST(−LAN)]
TSC	–							0.830	0.836
TY	ns	–						0.745	0.707
TSY	0.361***	0.902***	–					0.762	0.730
TN	0.163**	0.452***	0.459***	–				0.728	0.743
TW	−0.177**	0.370***	0.370***	−0.350***	–			0.723	0.688
PM_1_	−0.212**	−0.379***	−0.439***	−0.418***	ns	–		–	_
PM_2_	−0.248*	ns	−0.215*	−0.249*	ns	0.510***		–	_

*ns* not significant; * 0.05 > *p* > 0.01, ** 0.01 > *p* > 0.001, *** *p* < 0.001

### Population structure

**Fig. 2:**
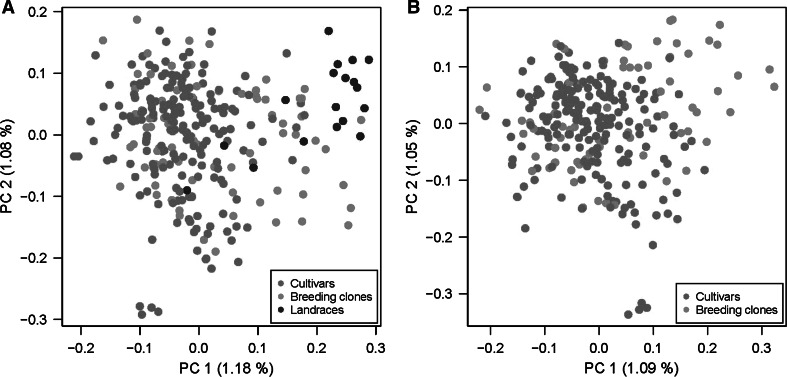
Principal coordinate plots of the QUEST population including (**a)** and excluding (**b**) the landraces, based on 183 alleles at 29 microsatellite loci. Genotypes were separated by the first two principal coordinates (PC) which were calculated on the basis of Jaccard’s distances. Numbers in parentheses are the percentage of explained variance by the PC

Jaccard’s distances were calculated based on the marker information of 183 microsatellite alleles at 29 loci, once for the full population (*n* = 282) and a second time only for cultivars and breeding clones (*n* = 264). Two microsatellite alleles were exclusively present in the landraces, and 31 microsatellite alleles were absent in the landraces, 25 of those with an overall allele frequency of less than 5 %. Principal coordinate analysis of the full population (Fig. 2a) showed that the landraces tended to cluster separately from cultivars and breeding clones, which formed a large cluster with the exception of four cultivars [cvs Arrow, Kennebec, Ramses and L 37(4×)] that formed a separate group. The variance explained by the first two principal coordinates was low, 1.18 % by PC1 and 1.08 % by PC2. The total explained variance of the first eleven coordinates was 10 %. Figure 2b shows the result of the principal coordinate analysis of the cultivars and breeding clones without the landraces, where PC1 and PC2 together explained 3.04 % of the variance. Principal coordinates one to eleven were extracted for association analysis.

Bayesian clustering indicated that *K* = 2 was the most likely number of subpopulations in the QUEST population. The landraces were most prominently represented in subpopulation 2 (Online Resource 3).

Potato genotypes can be grouped according to cytoplasm type, some of which show correlation with agronomic traits (Sanetomo and Gebhardt 2015). We therefore genotyped the QUEST population for six cytoplasm types according to (Hosaka and Sanetomo 2012). The majority of the cultivars, 144 of 191, had T type cytoplasm, the most prevalent type in *S. tuberosum* ssp. *tuberosum*. D type cytoplasm from *S. demissum* and W type cytoplasm from various wild potato species was present in similar numbers in varieties (D = 23 and W = 22), breeding clones (D = 12 and W = 15) and landraces (D = 1 and W = 1). 27 of 38 W cytoplasm types showed the W/γ sub-type of *S. stoloniferum*. The A type cytoplasm (prevalent in *S. tuberosum* spp. *andigena*) was found in two varieties (Gorbea and Kasta) and in seven landraces. P type cytoplasm from *S. phureja* and M type (Mother type, or an ancestral type of Andean cultivated potatoes) were rare (P = 6 and M = 1) and occurred only in landraces. The cytoplasm type was put into context with the inferred subpopulations from the Bayesian clustering approach. 90.6 % of T type and 86.6 % of W type cytoplasm was present in subpopulation 1. A, M and P type cytoplasm were solely present in subpopulation 2, while the D type cytoplasm was equally distributed in both inferred subpopulations 1 and 2 (52.8 and 47.2 %, respectively) (Table 3).

**Table 3:** Cytoplasm types in the QUEST population and their distribution in the K = 2 subpopulations inferred by Bayesian clustering

Cytoplasm type	Total	Subpopulation		% per subpopulation	
		1	2	1	2
T	192	174	18	90.6	9.4
D	36	19	17	52.8	47.2
W	38	33	5	86.6	13.2
A	9	–	9	–	100
P	6	–	6	–	100
M	1	–	1	–	100
Total	282	226	56		

### Marker–trait associations

A total of 416 DNA polymorphisms at 48 loci were tested for association with TSC, TY, TSY, TW, and TN in the QUEST population: 221 bi-allelic SNPs and 6 indels scored in amplicon sequences of candidate genes (Online Resource 4), 8 allele-specific PCR markers and 181 microsatellite alleles. In addition, the epistatic interaction between the PCR markers *Pain1*-*8c* and *Rca*-*1a* (Li et al. 2010) as well as the cytoplasm type were tested for associations with the tuber traits. Three association analyses were performed. The QUEST population with (+LAN) and without (−LAN) the landraces was analysed with a mixed linear model including kinship and population structure (MM-PK model). In addition, the QUEST(−LAN) population was analysed with a simple model without correction for population structure (GLM) (Table 4). With all models, the highest number of associations was obtained for TSC. Except TSY, the number of marker–trait associations dropped as expected when applying the MM-PK model (−LAN) instead of the GLM model (−LAN). Excluding markers with a minor allele frequency (MAF) of less than 1 % also eliminated several associations. Excluding the landraces from the association analysis further reduced the number of marker associations with TY and TSY and eliminated all associations with TN and TW. In accordance with the similar phenotypic distribution of TSC in cultivars, breeding clones and landraces (Fig. 1), associations with TSC were least sensitive against the removal of landraces from the population (Table 4). Twenty-two, two and five marker associations with TSC, TY, and TSY, respectively, were consistently detected in the QUEST population with or without the landraces when applying the MM-PK model.

**Table 4:** Number of marker–trait associations (MAF ≥1 %) detected at *p*-values <0.05, <0.01, and <0.001 with the models GLM, MM-PK with (+LAN) and without landraces (−LAN)

Trait	No. of markers (*p* < 0.05)				No. of markers (*p* < 0.01)				No. of markers (*p* < 0.001)			
	GLM (−LAN)	MM-PK (+LAN)	MM-PK (−LAN)	Persistent in MM	GLM (−LAN)	MM-PK (+LAN)	MM-PK (−LAN)	Persistent in MM	GLM (−LAN)	MM-PK (+LAN)	MM-PK (−LAN)	Persistent in MM
TSC	104 (109)	66 (71)	56 (61)	44 (48)	47 (49)	29 (31)	28 (31)	22 (23)	24 (26)	11 (11)	10 (10)	10 (10)
TY	41 (46)	26 (28)	21 (23)	13 (15)	13 (14)	5 (7)	2 (3)	2 (3)	2 (2)	1 (1)	1 (1)	1 (1)
TSY	29 (30)	46 (49)	29 (31)	24 (26)	3 (4)	11 (12)	8 (9)	5 (6)	1 (1)	2 (2)	2 (2)	2 (2)
TN	32 (34)	30 (38)	0	0	7 (8)	16 (21)	0	0	3 (4)	2 (5)	0	0
TW	60 (63)	38 (40)	0	0	24 (24)	8 (9)	0	0	4 (4)	1 (1)	0	0

The number of marker–trait associations when including markers with MAF <1 % are shown in parenthesis

Of 19 candidate loci tested, 14 had previously shown associations with tuber traits in the CHIPS-ALL population (Draffehn et al. 2010; Fischer et al. 2013; Li et al. 2005, 2008; Schreiber et al. 2014). DNA polymorphisms at five of these loci were associated at *p* < 0.01 with TSC and in some cases with TSY in the QUEST(−LAN) population (*n* = 264) when using the MM-PK model and excluding markers with MAF < 1 % (Table 5). The SNP allele *AGPaseS_C*_*1612*_ was associated with decreased TSC (negative effect) as previously found in the CHIPS-ALL population (Schreiber et al. 2014). The allele-specific PCR marker *Pain1*-*8c* and the SNP allele *LapN_A*_*2746*_ both had a positive effect on TSC and TSY in the QUEST(−LAN) population as they did in the CHIPS-ALL population (Draffehn et al. 2010; Fischer et al. 2013; Li et al. 2008, 2013). The negative effect of the *InvCD141*-*Sa* allele and the corresponding SNP haplotype *InvCD141_A*_*280*_*T*_*288*_*T*_*339*_*T*_*543*_*A*_*630*_ (Draffehn et al. 2010; Schreiber et al. 2014) on TSC was reproducible in the QUEST(−LAN) population as well as the positive effect of *LapN_C*_*3117*_ (Fischer et al. 2013). The *Rca*-*1a* marker that negatively affected chip quality (corresponding to tuber reducing sugar content which is inversely correlated with TSC) in the CHIPS-ALL population (Li et al. 2008) decreased TSC in the QUEST(−LAN) population. The interaction between *Rca*-*1a* and *Pain1*-*8c* for TSC and TSY observed in the CHIPS-ALL population (Li et al. 2010) was not detected in the QUEST population. The SNP haplotype *PWD_A*_*10916*_*T*_*10923*_ associated with decreased TSC in the QUEST(−LAN) population was not detected before in the CHIPS-ALL population, where different SNPs in the same amplicon were associated with increased TSC and TSY (Schreiber et al. 2014).

**Table 5:** Marker–trait associations in the QUEST population without landraces (−LAN) based on the mixed model MM-PK

Marker	Marker alleles^a^	Locus position (Mbp)	Allele frequency^b^	TSC (*R* ^2^)	TY (*R* ^2^)	TSY (*R* ^2^)	PM_1_ (*R* ^2^)
*AGPaseS_snp1612*	T/C	Chr01:86.09	0.095 (C)	**(3.6) **↓**	ns	ns	ns
*SSIV_snp2679*	A/T	Chr02:30.14	0.063 (T)	ns	**(3.7) ↑	*(2.0) ↑	ns
*SSIV_snp2786*	T/A		0.041 (T)	ns	ns	ns	**(4.5) **↓**
*Pain1*-*8c*	0/1	Chr03:39.25	0.155 (1)	***(4.5) ↑	ns	**(3.1) ↑	ns
*PGM1*-*3_snp440*	C/T		0.195 (T)	ns	ns	ns	**(4.4) **↑**
*PGI1*-*4_snp333*	A/G	Chr04:64.78	0.033 (G)	**(2.4) ↑	ns	ns	ns
*PGI1*-*4_snp303* ^c^	A/G		0.092 (G)	**(2.5) ↓	ns	ns	ns
*PGI1*-*4_snp267* ^c^	G/A		0.086 (A)	**(3.0) ↓	ns	ns	ns
*PGI1*-*4_snp252* ^c^	T/A		0.087 (A)	**(3.1) ↓	ns	ns	ns
*PGI1*-*4_snp235* ^c^	C/T		0.115 (T)	**(2.9) **↓**	ns	ns	ns
*PGI1*-*4_indel202*	0/1		0.322 (1)	**(2.7) ↓	ns	ns	ns
*StCDF1_snp1812*	A/T	Chr05:04.54	0.157 (T)	ns	***(4.3) ↑	***(4.9) ↑	ns
*StCDF1_snp1795*	C/A		0.074 (A)	ns	ns	ns	**(4.4) **↓**
*StBEL5_snp2961*	T/C	Chr06:54.71	0.055 (C)	ns	ns	ns	**(5.5) ↑
*PWD_snp10916* ^d^	C/A	Chr09:60.56	0.300 (A)	**(2.5) ↓	ns	ns	ns
*PWD_snp10923* ^d^	C/T		0.302 (T)	**(2.4) ↓	ns	ns	ns
*Rca*-*1a*	0/1	Chr10:50.94	0.462 (1)	**(2.4) ↓	ns	ns	ns
*STM1106*-*b (InvCD141)*	0/1	Chr10:55.85	0.112 (1)	***(4.1) ↓	ns	ns	ns
*InvCD141*-*Sa* ^e^	0/1	Chr10:55.85	0.665 (1)	***(6.6) ↓	ns	ns	ns
*InvCD141_snp280* ^e^	G/A	Chr10:55.85	0.135 (A)	***(10.0) ↓	ns	*(2.1) ↓	ns
*InvCD141_snp288* ^e^	C/T		0.168 (T)	***(6.7) ↓	ns	ns	ns
*InvCD141_snp339* ^e^	C/T		0.148 (T)	***(10.2) ↓	ns	ns	ns
*InvCD141_snp378*	C/T		0.214 (T)	**(3.1) ↑	ns	ns	ns
*InvCD141_snp426*	T/C		0.197 (C)	***(9.7) ↓	ns	*(2.2) ↓	ns
*InvCD141_snp543* ^e^	C/T		0.147 (T)	***(9.6) ↓	ns	*(2.1) ↓	ns
*InvCD141_snp630* ^e^	G/A		0.138 (A)	***(6.6) ↓	ns	ns	ns
*STM0037*-*g*	0/1	Chr11:08.21	0.814 (1)	**(2.5) ↓	ns	**(3.4) ↓	ns
*LapN_snp2746*	G/A	Chr12:02.34	0.092 (A)	***(4.4) ↑	*(1.6) **↑**	***(4.2) ↑	ns
*LapN_snp3117*	T/C		0.181 (C)	**(3.0) ↑	ns	ns	ns
Cytoplasm type	–	–	–	**(3.4)	ns	**(3.3)	ns

Markers with minor allele frequency ≥1 % and at least one association at *p* < 0.01 are shown

*ns* not significant at α = 0.05; * significant at 0.01 ≤ *p* < 0.05, ** significant at 0.001 ≤ *p* < 0.01, *** significant at *p* < 0.001; Arrows indicate the direction of the effect of presence of the microsatellite or PCR marker allele or the effect of increasing dosage of the minor frequency SNP allele on the trait: ↑ increasing, **↓** decreasing mean values for TSC, TY, TSY, and PM_1_. Numbers in **bold** indicate markers that were persistent in the two association models MM-PK (+LAN) and MM-PK (−LAN) at *p* < 0.01)

^a^The nucleotide present in the reference potato genome sequence (*S. phureja*) is on the left position, the alternative allele (*S. tuberosum*) on the right

^b^For SNPs the minor allele frequency (MAF) is shown with the minor frequency SNP allele in parenthesis; MAF was calculated including the allele dosage. The ‘allele’ frequency of microsatellite and allele-specific PCR markers corresponds to the frequency of the presence (1) of the marker allele without counting allele dosage

^c^The four *PGI*-*4* SNPs are in nearly complete LD and form a haplotype

^d^The two *PWD* SNPs are in nearly complete LD and form a haplotype

^e^The six *InvCD141* SNPs are in nearly complete LD and form a haplotype, the allele-specific marker *InvCD141*-*Sa* detects this haplotype

**Fig. 3:**
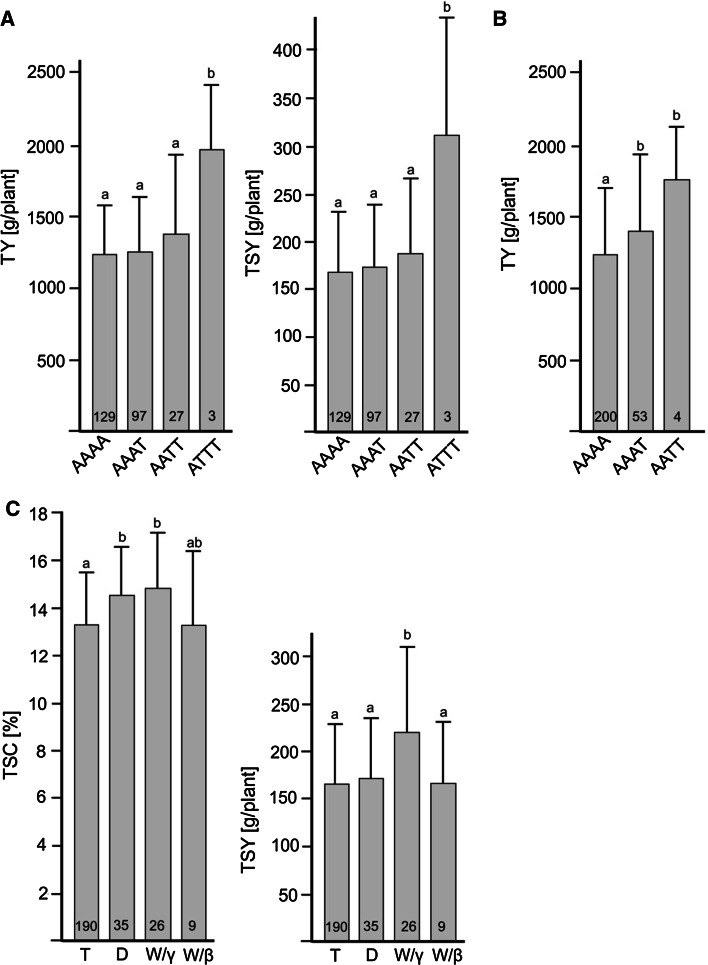
Phenotypic effects of genotype classes of SNPs *StCDF1_snp1812* (**a**), *SSIV_snp2679* (**b**), and cytoplasm type (**c**) in the QUEST(−LAN) population. Means (*grey bars*) and standard deviations of TSC, TY, and/or TSY are shown. Significant differences between genotype classes are indicated by *a* and *b* (ANOVA post hoc test LSD, *p* < 0.05). The number of individuals in each genotype class is indicated at the bottom of the bar. The three genotypes with the highest dosage (*ATTT*) of the allele *StCDF1_T*_*1812*_ associated with increased TY and TSY were the variety Isla and the breeding clones 2001Q29-10 and 2003P54-4. The four genotypes with the highest dosage (*AATT*) of the allele *SSIV_T*_*2679*_ associated with increased yield were the varieties Murato, Melody, Riviera, and Opal (Online Resource 1)

SNPs in three of five new candidate genes were associated at *p* < 0.01 with TSC, TY, and TSY in the QUEST(−LAN) population (Table 5). Most significant (TY: *p* = 6.77E–4, TSY: *p* = 3.44E–4) was the SNP allele *StCDF1_T*_*1812*_ that strongly increased average TY and TSY when present in at least triplex allele dosage (Fig. 3a). This SNP is not identical to the single SNP in the *StCDF1* gene that was strongly associated with plant maturity in a variety panel of 83 genotypes (Kloosterman et al. 2013) (Christian Bachem, Wageningen University, personal communication). The low frequency SNP allele *SSIV_T*_*2679*_ had a dosage dependent positive effect mainly on TY (Fig. 3b) (*p* = 1.53E–3). The DNA variants in the *PGI1*-*4* gene were mainly associated with TSC (*p* = 2.15E–3–7.09E–3).

**Fig. 4:**
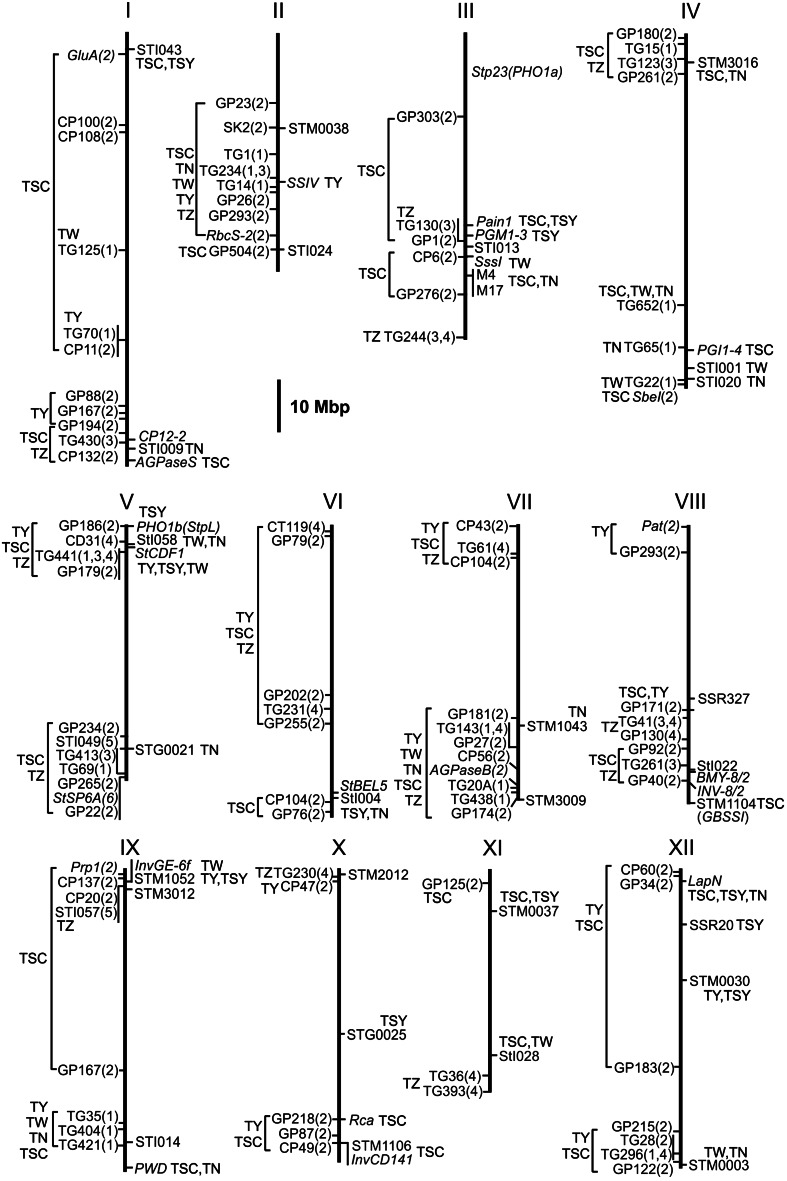
Physical maps of the 12 potato chromosomes, showing to the right of each chromosome the genomic positions of the markers genotyped in the QUEST population, and to the left markers linked to previously mapped QTL for tuber starch content (TSC, specific gravity (SG) in Bonierbale et al. 1993), yield (TY), starch yield (TSY), tuber weight (TW), tuber number (TN), and tuberization (TZ, in vitro tuberization (ivt) and greenhouse tuberization (gt) in Šimko et al. 1999). *Numbers in parenthesis* after the markers are numerical codes for the corresponding reference: 1 (Bonierbale et al. 1993), 2 (Schäfer-Pregl et al. 1998), 3 (van den Berg et al. 1996), 4 (Šimko et al. 1999), 5 (Zhou et al. 2014), and 6 (Navarro et al. 2011). RFLP marker sequences were retrieved from the databases Sol Genomics Network (TG, CT, and CD markers, http://www.sgn.cornell.edu/) and GABI Primary Database (GP and CP markers, GluA, SK2, SbeI, AGPaseB, pat, prp1, http://www.gabipd.org/). Microsatellite primer sequences were retrieved from the literature cited in Materials and Methods. Gene sequences were obtained from GenBank entries (http://www.ncbi.nlm.nih.gov/). Sequences were BLASTed against the potato genome sequence (pseudomolecules v4.03, http://potato.plantbiology.msu.edu/blast.shtml). QTL are shown red next to the linked or associated marker locus or next to the *brackets* indicating genome segments harbouring the QTL. Loci associated with TSC, TY, TSY, TW, and/or TN in the QUEST(+LAN) population but not in the QUEST(−LAN) population (MM-PK model, *p* < 0.01, MAF >1 %, see Online Resource 5) are shown in *blue letters*. Loci associated with TSC, TY, and/or TSY in both the QUEST(−LAN) and QUEST(+LAN) population are shown in *green letters* (see Table 5). Genes with known function are in *italics*

Alleles at 8 of 29 microsatellite loci showed putative associations with TSC and/or TSY at *p* < 0.01) (Fig. 4). Three of these microsatellites are located in candidate genes: *STM1104* in granule bound starch synthase I (*GBSSI,* chromosome VIII), *STM1052* in the tandem duplicated apoplastic invertase genes *InvGE* and *InvGF* (chromosome IX) and *STM1106* in the apoplastic invertase gene *InvCD141* (chromosome X). Four microsatellites are located in loci encoding putative transcription factors (*STI043*, PGSC0003DMG400016379; *STI004*, PGSC0003DMG400003372), an oxidoreductase/transition metal ion binding protein (*STG0025*, PGSC0003DMG400028767) and an unknown gene (*STI028*, PGSC0003DMG400007365). The fifth microsatellite *STM0037* is located in an intergenic region on the same superscaffold (PGSC0003DMB000000133) as the sucrose transporter *Sut1* (PGSC0003DMG400009213) and a putative invertase or pectinesterase inhibitor (PGSC0003DMG400038811) (Online Resource 2). The negative effect of the frequent allele *STM0037*-*g* on TSC and TSY (Table 5) has been previously detected in the CHIPS-ALL population (Li et al. 2008).

Cytoplasm type was associated with TSC and TSY. Genotypes with cytoplasm type W/γ had the highest means of TSC and TSY compared with cytoplasm types T, D and W/β (Fig. 3c). This positive effect of W/γ cytoplasm on TSC has been observed before in three independent association mapping populations (Sanetomo and Gebhardt 2015).

Association analysis (MM-PK model) for plant maturity based on 154 genotypes of the QUEST population comprising only varieties and breeding clones (PM_1_ data set) resulted in six marker–trait associations at 0.001 < *p* < 0.01 at six independent loci. The six markers, four SNPs in genes *SSIV, PGM1*-*3, StCDF1* and *StBEL5* (Table 5) and two microsatellite alleles (*STM1104*-*b, STG0025*-*b*), were not significantly associated with the tuber traits. Association analysis based on 90 varieties (PM_2_ data set) resulted in two different putative associations at 0.001 < *p* < 0.01 of SNP *SssI_snp6001* and microsatellite allele *STI022*-*e* (not shown).

The results of the association analysis in the QUEST(+LAN) population (*n* = 280) with the MM-PK model under liberal stringency criteria (*p* < 0.05, markers with MAF <1 % included) are shown in Online Resource 5. Additional putative marker–trait associations with small effects were detected for TSC, TY, and TSY, some of which were reproducible when compared to the CHIPS-ALL population. For example, the haplotype *AGPaseS_T*_*1411*_*C*_*1457*_ associated with increased TSC (Schreiber et al. 2014) was detected under these conditions. It was present with less than 1 % frequency in the QUEST population. Moreover, 20 markers at 16 loci (7 candidate and 9 microsatellite loci) on chromosomes I, III, IV, V, VI, VII, IX, X, and XII were associated with tuber number at *p* < 0.01, five of which with MAF <1 %. In 19 of 20 cases the minor frequency allele was associated with higher tuber number. The most significant associations with TN (*p* < 0.001) were detected by the microsatellite alleles *STI058*-*g* on chromosome V and *STM1043*-*d* on chromosome VII. Whilst *STI058* is located in a non-annotated gene, *STM1043* is located in the sucrose synthase gene *Sus*-*7/2* (*Sus3*, PGSC0003DMG400013546). Nine markers at seven loci (three candidate and four microsatellite loci) on chromosomes III, IV, V, IX, XI, and XII were associated with tuber weight at *p* < 0.01, one with MAF <1 %. In six of the nine cases the minor frequency allele was associated with higher tuber weight. One of those was *StCDF1_T*_*1812*_, the only SNP allele that was associated with increased TW as well as increased TY and TSY. The strongest association with TW (*p* < 0.001) showed the microsatellite allele *STI028*-*f* on chromosome XI that is located in an unknown gene (PGSC0003DMG400007365). Little overlap was observed between markers associated with TW and TN. Three markers were associated with both TW and TN, two however only at *p* < 0.05 and one at *p* < 0.01. The SNP allele *SssI_C*_*5907*_ had a positive effect on both TW and TN, whereas the SNP allele *LapN_G*_*2783*_ and the microsatellite allele *STM0003*-*d* were both associated with lower TW and higher TN.

Figure 4 gives a graphical overview of the positions on the potato physical map of the loci tested for association and the results of the association analysis in the QUEST population with *p* < 0.01 and excluding markers with MAF <1 %.

### Linkage disequilibrium between SNPs

LD was calculated between all pairs of 220 bi-allelic SNP loci scored in the QUEST(+LAN) population (Online Resource 6). The highest LD was observed among SNPs within the same amplicon, which allowed the identification of several haplotypes involving 2–12 SNPs.

## Discussion

### Comparison of QUEST versus CHIPS-ALL population

The CHIPS-ALL population where diagnostic markers for TSC, TSY, and TY have been originally discovered, was composed of 14 % cultivars, mostly German varieties, and 86 % breeding clones (Li et al. 2008). The QUEST population was assembled from 68 % cultivars, the majority from The Netherlands, Spain, Germany and Austria, 26 % clones from breeding programs of APPACALE in Northern Spain and CIP in Peru, and 6 % Andean landraces (Online Resource 1). Tuber starch content (TSC), yield (TY), weight (TW), number (TN), and starch yield (TSY) were evaluated in the QUEST population in a moderate Mediterranean climate at 42° North under shorter day length compared to the CHIPS-ALL population, which has been evaluated between 53° and 54° North under long days in the Atlantic climate of Northern Germany. The cultivation period was similar. The high positive correlations of TSC and TY with TSY were similar to the CHIPS-ALL population. These correlations are expected as TSY is derived from TSC and TY. Also the positive correlation of TY with TW and TN is not surprising as tuber weight and number are the constituents of tuber yield. A similar correlation was obtained between tuber yield and size in a QTL linkage mapping experiment conducted in a tetraploid F1 family (Bradshaw et al. 2008). The negative correlation between TW and TN illustrates the sink–sink competition among developing tubers (Struik 2007). Increase of tuber weight is compensated by lower tuber numbers and vice versa. Plant maturity has not been evaluated in the CHIPS-ALL population and in only about half of the QUEST population. The correlations between PM, TSC, TY, and TSY illustrate the fact that longer vegetation periods (later maturity) can have a positive effect on tuber starch accumulation and yield components. Similar correlations were reported in independent association mapping panels (D’hoop et al. 2011; Urbany et al. 2011).

### Comparison of cultivars and breeding clones versus landraces

Only minor differences were observed for the phenotypic means between cultivars and breeding clones, whereas the Andean landraces were clearly different, having lower average TY, TW, TN, and TSY but not TSC. Reasons for this can be the lack of climatic adaptation and the diploidy of some genotypes in the landrace group such as *S. phureja*. The landraces were cultivated potato species and subspecies other than *S. tuberosum* ssp. *tuberosum* such as *S. tuberosum* ssp. *andigenum*, *S. phureja, S. stenotomum, S. ajanhuiri*, and *S. goniocalyx* (Online Resource 1). At the genotypic level, the landraces also separated from cultivars and breeding clones, both in principal coordinate analysis and Bayesian clustering, however not as clear cut as diploid *S. tuberosum* ssp. *tuberosum* breeding clones and wild potato species were separated from tetraploid cultivars (Hamilton et al. 2011; Stich et al. 2013). The overall substructure of the QUEST population was moderate and even more uniform when the landraces were excluded [QUEST(−LAN)] compared with the complete population [QUEST(+LAN)]. This result is in line with previous structure analyses in populations of tetraploid potato (D’hoop et al. 2010; Hamilton et al. 2011; Li et al. 2008; Pajerowska-Mukhtar et al. 2009; Urbany et al. 2011).

Based on the phenotypic distributions and population structure, we decided to analyse marker–trait associations in both the QUEST(−LAN) and QUEST(+LAN) population. Marker–trait associations without the landraces should better reflect the situation in advanced cultivars of tetraploid potato, whereas marker–trait associations including the landraces might point to alleles that play a role when the phenotypic distribution is broadened toward the low value end.

### Reproducibility of marker–trait associations

This is the first report on the reproducibility of diagnostic DNA markers for complex potato tuber traits in an independent association panel that was field evaluated in a different geographic region. Marker–trait associations that can be reproduced under such conditions have added value for breeding applications.

When applying the MM-PK model to the QUEST(−LAN) population, excluding markers with *p* > 0.01 and MAF <1 % (Table 5), five alleles of the candidate genes *AGPaseS, Pain1, InvCD141,* and *LapN* and one allele of microsatellite *STM0037* showed the same effects as in the CHIPS-ALL population, although the amount of total variance explained by these alleles were generally lower. On the contrary, eight loci with markers diagnostic in the CHIPS-ALL population did not show associations in the QUEST population under the above conditions. In some cases SNPs scored in the CHIPS-ALL population were not detected or not scorable in the QUEST population. In other cases however, the same markers were polymorphic and scored in both populations. As examples we discuss markers *Stp23*-*8b(PHO1a*-*H*_*A*_*), StpL(PHO1b)*-*3b,* and *StpL(PHO1b)*-*3e* detecting specific alleles of the starch phosphorylase genes *Pho1a* and *Pho1b* on chromosome III and V, respectively, which were highly significant associated with TSC and TSY in the CHIPS-ALL population (Li et al. 2008). In particular, the marker *Stp23*-*8b(PHO1a*-*H*_*A*_*)* has been validated by marker-assisted selection in a German breeding population (Li et al. 2013). Moreover structural and functional analysis suggested that the *PHO1a*-*H*_*A*_ allele might directly contribute to the variation of tuber starch content. Presence of *PHO1a*-*H*_*A*_ reduced overall starch phosphorylase activity in tubers, which could lead to increased tuber starch content by means of limitation of starch breakdown (Schreiber et al. 2014). Compared to the CHIPS-ALL population, the frequency of markers *Stp23*-*8b(PHO1a*-*H*_*A*_*)* and *StpL(PHO1b)*-*3b* was lower in the QUEST population (17 % versus 27 % and 24 % versus 50 %, respectively), whilst the frequency of marker *StpL(PHO1b)*-*3e* was the same (53 %). Reduced allele frequencies, diminished LD with the effect causing loci and/or genotype by environment interactions are likely responsible for not observing any more these and other marker–trait associations in the QUEST population. Comparable results were obtained when marker–trait associations were analysed in three different rice panels (Zhang et al. 2014).

### Novel diagnostic markers

From the SNPs scored in new candidate genes, the SNPs *SSIV_snp2679* and *StCDF1_snp1812* were particularly interesting because they were the only markers that were associated with tuber yield and starch yield but not with tuber starch content (Table 5, Online Resource 5). Such marker–trait associations were not identified previously. They represent the tip of an iceberg though, as they explained only a small fraction of the genetic variation of yield and starch yield in the QUEST population. The minor frequency SNP alleles *SSIV_T*_*2679*_ (MAF = 6.3 %) and *StCDF1_T*_*1812*_ (15.7 %) increased mean TY and TSY. The effect depended on the allele dosage. *StCDF1_snp1812* was not associated with plant maturity, whereas *StCDF1_snp1795* was associated with plant maturity but not with tuber yield. Both SNPs showed no LD with each other (Online Resource 6). This suggests that the beneficial yield allele detected by *StCDF1_T*_*1812*_ is independent from the plant maturity QTL.

Under liberal conditions for the association test, additional putative associations of minor frequency alleles with positive effects on TY and/or TSY were detected in the QUEST(+LAN) population (Fig. 4, Online Resource 5). Some of these alleles were rare (MAF < 3 %) (e.g., *SSSI_A*_*6015*_*, STM1043*-*d(Sus*-*7/2), STG0025*-*d, InvCD141_T*_*481*_). Provided validation is warranted, such alleles provide new opportunities for breeding applications. The generation of parental and F1 populations with high frequencies of positive alleles for TY and TSY by marker assisted selection is expected to increase precision and accelerate the development of cultivars with high starch yield.

Some QTL for tuber number and for tuber weight were identified by association genetics in the QUEST(+LAN) population. The majority of the minor frequency alleles had positive effects on TW and TN. The absence of these associations in the QUEST(−LAN) population indicates that the loci underlying these QTL have only small or no effect in advanced germplasm. Nevertheless, enrichment of low frequency SNP alleles associated with increased TW and TN such as *SSSI_C*_*5907*_ (frequency 4.6 %) or the combination of microsatellite alleles *STI058*-*g* (2.9 % frequency, higher mean TN,) and *STI058*-*b* (40.9 %, higher mean TW) might help to accelerate yield improvement when introgressing, for example, resistance traits from low yielding exotic germplasm.

Besides nuclear DNA polymorphisms, DNA variation in plastid and mitochondrial DNA (cytoplasm type) is also relevant for TSC and TY (Sanetomo and Gebhardt 2015). This was confirmed by the association of cytoplasm type with TSC and TSY in the QUEST population. This is conceivable when considering that plastidic and mitochondrial encoded genes have important roles in carbon fixation and energy metabolism. The W/γ cytoplasm type improved mean starch yield by approximately 30 % compared with T, D and W/β cytoplasm types (Fig. 3). As with other beneficial markers, the frequency of W/γ cytoplasm type was with 10 % rather low in varieties and breeding clones. The targeted combination of W/γ cytoplasm type with nuclear markers diagnostic for increased starch yield should facilitate the development of cultivars for industrial starch production.

### Marker associations with plant maturity

Potatoes in their original habitat in the Andes are short day adapted plants and require short photoperiods for tuberization. Plant maturity is a phenotype that is connected with the plant’s ability to tuberize under long day conditions as present in middle and Northern European late spring and summer. Tuberization under short day conditions is controlled by the wild type allele *StCDF1.1* at the *StCDF1* locus on potato chromosome V, whereas the defective *StCDF1.2* and *StCDF1.3* alleles induce tuberization in long days (Kloosterman et al. 2013). We were not able to detect the ‘late’ *StCDF1.1* allele and the ‘early’ *StCDF1.2* and *StCDF1.3* alleles in the QUEST population, either due to technical difficulties in developing a suitable PCR marker assay or because these alleles from diploid germplasm were absent in the tetraploid QUEST population. Instead, we genotyped the QUEST population for 16 novel SNPs within the *StCDF1* coding region, which can theoretically discriminate 2^16^ haplotypes. At least eight haplotypes were distinguishable in the QUEST population (Online Resource 6). None of the 16 SNPs was associated with a major effect on PM as was detected in a panel of 83 varieties by a single SNP in the *StCDF1* gene itself and several SNPs separated by 130 kb from the *StCDF1* locus (Kloosterman et al. 2013). The size of the effect of the associated SNP *StCDF1_snp1795* in the QUEST population was in the same order of magnitude as the other, independent markers associated with PM_1_. We cannot exclude that we missed SNPs associated with a major effect *StCDF1* allele or that the phenotypic data were insufficient. Alternatively, the *StCDF1* locus might be less influential on natural variation of plant maturity when the plants are cultivated in a geographical region with shorter day length during the growing season such as Northern Spain. The length of the vegetation period recorded by breeders as plant maturity or ‘earliness’ is certainly a polygenic trait that is influenced by additional developmental processes and additional loci as demonstrated in previous linkage and association mapping studies for plant maturity, reviewed in (Gebhardt et al. 2014).

### Comparison with QTL for tuber traits mapped in experimental populations

QTL for specific gravity (corresponding to TSC), tuber yield, weight per tuber and tuber number have been mapped by linkage analysis in three tetraploid families by means of RFLP (restriction fragment length polymorphism) markers (Bonierbale et al. 1993). Schäfer-Pregl and colleagues (1998) mapped QTL for tuber starch content and yield in two diploid families also using RFLPs (Schäfer-Pregl et al. 1998). Furthermore, two QTL each for tuber size and yield were mapped in a tetraploid F1 family via linkage to AFLP (amplified fragment length polymorphism) markers (Bradshaw et al. 2008). Tuber number might correlate with stolon number and thereby with tuberization (TZ) that is the photoperiod dependent induction of tubers in the stolon tips. QTL linkage studies for tuberization were conducted with RFLP markers in two interspecific, diploid families (Šimko et al. 1999; van den Berg et al. 1996) and with AFLP and some microsatellite markers in yet another tetraploid family (Zhou et al. 2014). AFLP markers unfortunately do not allow in silico comparison of QTL map positions across different mapping experiments due to the lack of sufficient sequence information, whereas the sequence information available for RFLPs, candidate genes and microsatellites linked or associated with QTL can be used to anchor the QTL to the potato physical map (Sharma et al. 2013). This allows to compare and integrate positional information from independent QTL mapping studies and to provide mutual validation for marker–trait associations and linkages (Fig. 4). Despite the fact that QTL linkage maps have low resolution and QTL linked markers can be physically distributed over most parts of a chromosome, the integration of five QTL linkage mapping experiments (Bonierbale et al. 1993; Schäfer-Pregl et al. 1998; Šimko et al. 1999; van den Berg et al. 1996; Zhou et al. 2014) with the marker–trait associations found in this study reveals some remarkable QTL ‘hot spots’ for tuber traits in the potato genome. We define here ‘hot spots’ as genomic regions, where markers linked with QTL for TSC, TY, TW, TN, TSY, and/or TZ in very different genetic backgrounds cluster and tag the same genome segment as associated markers. The most prominent hot spot comprises the distal segment of approximately five Mbp on the North arm of chromosome V, which harbours among other candidate genes (Schreiber et al. 2014) the *StCDF1* locus that controls photoperiod dependent tuberization (Kloosterman et al. 2013). Prolonged vegetation period (later plant maturity) is correlated with higher tuber starch content and yield (Urbany et al. 2011). The QTL for TY, TSC, TSY, TW, TN, and TZ observed in this region could be pleiotropic effects of *StCDF1* or caused by several, physically linked genes (Schreiber et al. 2014). Remarkable is also the distal region of 10 Mbp on the South arm of chromosome V, which contains genes controlling TSC, TN, and TZ. An excellent candidate in this region is the *StSP6* locus, which also controls tuberization (Navarro et al. 2011). Other examples for QTL hot spots are the 20 Mbp region around the *SSIV* locus on chromosome II and the distal 15–20 Mbp segments on the South arms of chromosomes IV, VII and VIII (Fig. 4).

Approximately half of all marker–trait associations were observed for microsatellite alleles and not candidate genes, particularly under liberal conditions for the association test (Online Resource 5). An unknown fraction of those are false positives, although some microsatellites were derived from candidate genes such as invertase, starch synthase, sucrose synthase and ‘tuber-specific and sucrose-responsive element binding factor’(Online Resource 2). On the other hand, the distribution of the markers linked with QTL for tuber traits on the physical chromosome maps (Fig. 4) suggests that these traits are controlled by multiple genes on virtually every chromosome arm. So that whatever ‘random’ markers are used for genotyping, some will be in sufficient LD with causal genes in order to show marker–trait association. To increase resolution and eventually identify with more accuracy the loci controlling complex tuber traits it will be necessary to substantially increase the marker density by genome wide association mapping, either using SNP genotyping arrays (Hamilton et al. 2011; Stich et al. 2013) or genotyping by sequencing (Elshire et al. 2011).

#### Author contribution statement

EMS performed the genotyping experiments, evaluated the data and drafted the manuscript; FO provided plant material and performed field experiments; LB, AA, and JIRdeG performed field experiments; J-CL, RS, and BW contributed to the genotyping experiments; ET conceived and coordinated the study; ER conceived and coordinated the study and provided plant material, CG conceived and coordinated the study and wrote the manuscript.

## Electronic supplementary material

Below is the link to the electronic supplementary material.

Supplementary material 1 (XLSX 48 kb)

Supplementary material 2 (DOCX 20 kb)

Supplementary material 3 (DOCX 37 kb)

Supplementary material 4 (DOCX 46 kb)

Supplementary material 5 (DOCX 55 kb)

Supplementary material 6 (XLSX 357 kb)
