# Supplementary material for: Identification and reproducibility of diagnostic DNA markers for tuber starch and yield optimization in a novel association mapping population of potato (Solanum tuberosum L.)
Source: Theor Appl Genet. 2016 Jan 29;129:767–85. doi: 10.1007/s00122-016-2665-7 (PMC4799268; doi:10.1007/s00122-016-2665-7)
Supplement: Supplementary file 2 — Supplementary material 2 (DOCX 20 kb) [file 122_2016_2665_MOESM2_ESM.docx]

**Identification and reproducibility of diagnostic DNA markers for tuber starch and yield optimization in a novel association mapping population of potato (*Solanum tuberosum* L.)**

E. M. Schönhals, F. Ortega, L. Barandalla, A. Aragones, J. I. Ruiz de Galarreta, J.-C. Liao, R. Sanetomo, B. Walkemeier, E. Tacke, E. Ritter, C. Gebhardt

Theoretical and Applied Genetics

Corresponding author: Christiane Gebhardt, Max-Planck Institute for Plant Breeding Research, Cologne, Germany ([gebhardt@mpipz.mpg.de](mailto:gebhardt@mpipz.mpg.de)).

**Online Resource 2**. SSR markers genotyped in the QUEST population with annotation, position, annealing temperature Ta, number of alleles scored per locus and reference.

| **SSR locus** | **Corresponding locus annotation** | **Pseudomolecule position (v4.03)** | **Ta (°C)** | **No. of scored alleles** | **Reference** |
| --- | --- | --- | --- | --- | --- |
| *STI043* | Zinc finger protein | Chr01:3506915..3507352 | 60-54 | 6 | Feingold et al. (2005) |
| *STI009* | RNA binding protein | Chr01:84890021..84896004 | 60-54 | 6 | Feingold et al. (2005) |
| *STM0038* | intergenic | Chr02:19628403..19628486 | 54 | 7 | Milbourne et al. (1998) |
| *STI024* | Hydroxyproline-rich glycoprotein family protein | Chr02:44529200..44537600 | 60-54 | 9 | Feingold et al. (2005) |
| *STI013* | fibrillarin homolog | Chr03:43661740..43664810 | 60-54 | 6 | Feingold et al. (2005) |
| *M4* | intergenic | Chr03:~49900000 | 55 | 4 | Odeny et al. (2010) |
| *M17* | intergenic | Chr03:49715572..49715401 | 56 | 6 | Odeny et al. (2010) |
| *STM3016* | intergenic | Chr04:6601695..6601715 | 60 | 7 | Milbourne et al. (1998) |
| *STI001* | Tuber-specific and sucrose-responsive element binding factor | Chr04:68721298..68722349 | 60-54 | 8 | Feingold et al. (2005) |
| *STI020* | BZIP domain class transcription factor | Chr04:70149220..70150660 | 60 | 6 | Feingold et al. (2005) |
| *STI058* | Not annotated | Chr05:4166571..4166620 | 60-54 | 7 | Feingold et al. (2005) |
| *STG0021* | Cornichon family protein | Chr05:45581656..45587896 | 55 | 6 | Ghislain et al. (2009) |
| *STI004* | Nucleic acid binding protein | Chr06:55860945..55863589 | 60-54 | 10 | Feingold et al. (2005) |
| *STM1043* | Sucrose synthase 2 | Chr07:40641700..40648100 | 53 | 5 | Milbourne et al. (1998) |
| *STM3009* | iojap protein | Chr07:56498441..56498586 | 64 | 2 | Milbourne et al. (1998) |
| *SSR327* | C2H2L domain class transcription factor | Chr08:35517211..35520484 | 55 | 6 | Frary et al. (2005) |
| *STI022* | Pseudo response regulator | Chr08:50038800..50047700 | 63 | 5 | Feingold et al. (2005) |
| *STM1104* | Granule-bound starch synthase 1, chloroplastic/amyloplastic | Chr08:56782190..56785790 | 57 | 4 | Milbourne et al. (1998) |
| *STM1052* | Apoplastic invertase | Chr09:2474700..2477140 | 59 | 5 | Milbourne et al. (1998) |
| *STM3012* | intergenic | Chr09:3964860..3964736 | 57 | 5 | Milbourne et al. (1998) |
| *STI014* | Cellulose synthase-like A1 | Chr09:55351420..55354606 | 60-54 | 6 | Feingold et al. (2005) |
| *STM2012* | Ethylene response factor ERF12 | Chr10:1122790..1121490 | 64 | 5 | Milbourne et al. (1998) |
| *STG0025* | Oxidoreductase/ transition metal ion binding protein | Chr10:33537359..33540698 | 55 | 5 | Ghislain et al. (2009) |
| *STM1106* | Apoplastic invertase | Chr10:55851950..55856810 | 60 | 10 | Milbourne et al. (1998) |
| *STM0037* | intergenic | Chr11:8213293..8213221 | 48 | 9 | Milbourne et al. (1998) |
| *STI028* | Conserved gene of unknown function | Chr11:37968820..37970010 | 60 | 7 | Feingold et al. (2005) |
| *SSR20* | Leucine-rich repeat-containing protein | Chr12:11259744..11265390 | 50 | 12 | Frary et al. (2005) |
| *STM0030* | intergenic | Chr12:22813885..22813902 | 53 | 7 | Milbourne et al. (1998) |
| *STM0003* | intergenic | Chr12:60055231..60055332 | 50 | 4 | Milbourne et al. (1998) |
