# Supplementary material for: Identification and reproducibility of diagnostic DNA markers for tuber starch and yield optimization in a novel association mapping population of potato (Solanum tuberosum L.)
Source: Theor Appl Genet. 2016 Jan 29;129:767–85. doi: 10.1007/s00122-016-2665-7 (PMC4799268; doi:10.1007/s00122-016-2665-7)
Supplement: Supplementary file 3 — Supplementary material 3 (DOCX 37 kb) [file 122_2016_2665_MOESM3_ESM.docx]

**Identification and reproducibility of diagnostic DNA markers for tuber starch and yield optimization in a novel association mapping population of potato (*Solanum tuberosum* L.)**

E. M. Schönhals, F. Ortega, L. Barandalla, A. Aragones, J. I. Ruiz de Galarreta, J.-C. Liao, R. Sanetomo, B. Walkemeier, E. Tacke, E. Ritter, C. Gebhardt

Theoretical and Applied Genetics

Corresponding author: Christiane Gebhardt, Max-Planck Institute for Plant Breeding Research, Cologne, Germany ([gebhardt@mpipz.mpg.de](mailto:gebhardt@mpipz.mpg.de)).


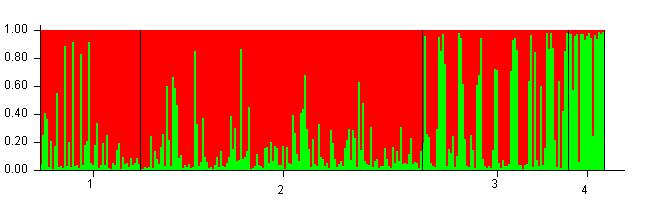


**Online Resource 3**: STRUCTURE graphical output of population structure for the QUEST population for subpopulations K=2. Barplot of inferred subpopulations K=2 are ordered according to genotype group: 1+2 = cultivars, 3 = breeding clones, 4 = landraces. Individual genotypes are plotted on the x-axis. The probability (*Q*-value) of each genotype belonging to subpopulation 1 (red) or 2 (green) is plotted on the y-axis.
