# Supplementary material for: Identification and reproducibility of diagnostic DNA markers for tuber starch and yield optimization in a novel association mapping population of potato (Solanum tuberosum L.)
Source: Theor Appl Genet. 2016 Jan 29;129:767–85. doi: 10.1007/s00122-016-2665-7 (PMC4799268; doi:10.1007/s00122-016-2665-7)
Supplement: Supplementary file 4 — Supplementary material 4 (DOCX 46 kb) [file 122_2016_2665_MOESM4_ESM.docx]

**Identification and reproducibility of diagnostic DNA markers for tuber starch and yield optimization in a novel association mapping population of potato (*Solanum tuberosum* L.)**

E. M. Schönhals, F. Ortega, L. Barandalla, A. Aragones, J. I. Ruiz de Galarreta, J.-C. Liao, R. Sanetomo, B. Walkemeier, E. Tacke, E. Ritter, C. Gebhardt

Theoretical and Applied Genetics

Corresponding author: Christiane Gebhardt, Max-Planck Institute for Plant Breeding Research, Cologne, Germany ([gebhardt@mpipz.mpg.de](mailto:gebhardt@mpipz.mpg.de)).

**Online Resource 4:** Amplicon sequences of candidate genes in the QUEST population including SNP positions, nucleotide alleles and identifications. Sequences were retrieved from pseudomolecules (v4.03) at <http://potato.plantbiology.msu.edu/cgi-bin/gbrowse/potato/>. Primer sequences are underlined. SNP numbering starts with A = 1 in the ATG initiation codon in the genomic locus PGSC0003DMG*********. SNPs highlighted yellow were scored in the QUEST population but not in the CHIPS-ALL population. SNPs highlighted grey were scored in the CHIPS-ALL population but not in the QUEST population. The remaining SNPs were scored in both populations.

**ADP-glucose pyrophosphorylase S, *AGPaseS* (PGSC0003DMG400000735)**

>Chr01:86095512..86094580

ggagatggatttgtcgaggtttgagactatacttttgaaattcttgaaggaaactggattagtcttgctagatatcacagctgagctaattaatatgagtaaatttgttggtcctccaagtgat

1259 1265

ttgttcacacaaaatctgtaacaatttcggatgatttttttcattgtt[t/C]ttctg[a/C

1268 1276 1284 1286

]tt[a/T]cttgctt[c/A]gttttac[t/C]g[a/G]ctgaaaggaattccgtcattaaac

1336 1348

aggtactagctgcaactcagacacc[c/T]ggggaagcagg[a/G]aaaaaatggtttcaag

1392 1411

gaacagcagatgctgttagaaaattta[t/C]atgggtttttgaggtttgt[c/T]tctaca

1426 1456 1457 1459

actcttta[a/G]gacacactaatccaccactgctcttctgt[t/C][g/C]a[c/T]ctga

1482 1501 1509

tataatttctgaaaaatg[a/T]ttcattattttttcctga[t/A]tcagtta[g/T]t

1511 1520 1529 1546 1547

[a/g]tgtacaga[t/C]atatagta[c/T]ttttgtgtttacaagg[t/C][g/A]accaa

1564 1592 1598

ttcttgcagga[c/T]gctaagaacaagaatattgaaaatatc[g/C]ttgta[c/t]tatc

1612

tggggatca[t/C]ctttataggatggattatatggagttggtgcaggtatgtccactttaa

1698

ctacctatgcactcctcattctttgttt[a/G]ctttttttttttttttttttaaacaacaaagtttctgactgcctccgatcttcaaacagaaccatattgacaggaatgctgatattactctttcatgtgcaccagctgaggacaggtttgtttaaattccaataagaaacaactagtaatggttaagtgaattgagtgagatcagaaacaatatggagaaagagtgcttctcctttaatattcgtatgcattacttcgaagtaaaaacatcaccacatttctttataccttattgtgattatgttaagctgagatttaagctttacttatcagccgagcatcagattttgggctggtca

**Calvin cycle protein CP12, *CP12-2* (PGSC0003DMG400009042)**

>chr01:62724275..62723829

ggcaacaattgctggtgttagtctcactagtccaaaattcttggccaaaaattccaactctc

71 76 84

ccaatt[c/t]taag[c/t]catttaa[g/a]ttcccatgccttaacaatccatggaaaaaa

117 142 150

tc[a/g]tcaactaaatttgggcttgtgtgt[g/a]cagcaac[a/g]ccagataacaagct

180

ttctgaccttgtggc[t/a]gatagtgttaagggggcagaggaagcgtgcaatgagaatccagctagtggagaatgcgcggcggcttgggatgttgtggaagaggcaagtgcagcggctagtca

300 327

tgcaagggacaagaa[g/t]aagcaggaagatgttttggaaaatta[t/c]tgcaaggaaaatccagagactgatgagtgccgtacatatgatagttgatactagttttgtgttatttaagcat

401(indel) 414 417

|tatgctttatttt[g/a]tt[c/a]ccaatttgaatcttgaatgctatgaattaggc

**Starch synthase IV, *SSIV* (PGSC0003DMG400008322)**

>chr02:30150096..30149228

Ctcaatgaagctcgtgtccatgcgttagaagaacttcaaaaagttcttggagagaaagaggatttgcatgggaagatcaatatcttagaaatgaagctagcagaaactgatgcacggctcagagttgcttcccaagaaaaaatacacgtggaacttctagaagaccagttggaaaagataaaaaatgaattgtctagcagcagaggcagtgaggaaaatgtgcttcatgtgaataattcagttcctctttcacatagtgattctgttaattcactgagtgaggaatttgattcgttaaggaaagaaaata

2565 2567

tattattgaagcaagatcttcaagcaatgaagtcag[g/a]g[c/a]ttagcttagtcaagg

2624

aaacagatgaacgcattttaatgctagagaaagaacgatca[g/a]ttcttgaatcttcttt

2662 2679

aagtgagttggaatctaaact[g/a]gctgcatctcaggaag[a/t]tgtttcagagctctc

2704 2706 2707 2719 2737

ttccttgaa[a/g]t[t/c][g/a]gagtgcaaaaa[a/t]ttatatgaaaaggtgga[a/g

2738 2755 2786

][c/t]acttacaagcgttgct[a/g]gctaaggcaaccaaacaagcagatcaagca[t/a]

2817

tatcagtgctacagcaaaaccaagagctgc[g/a]gaaaaaggttgacaggcttgaagaatctcttgaagaggctagcatctataaactttcatctgaaaaattacagcagtataatgaacaaatgcagcaaaagataaaacttcttgatgaacgccttcagaggtctgatgaagagatacaatct

3017

tatgtccagctgcatcaggattctgtcaaggagtttcaagatacactc[g/a]ataatttgaaaaatgaaactaaaaaaaaagctctagatgaacctgtaaacgagatgccttcggaattttg

**Phosphoglucomutase 1, *PGM1-3*  (not annotated)**

>Chr03:41510113..41509054

ATGGCTATGGAGAGTGCATTGACATCCACACGAGTTTCAATTCCATCGTTGTGTTCTGGGAT

93

CAGTTCATCTCATCATCACCATAGATCCTT[C/A/T]TCATTTCTCAATTTCCCCAAATTGT

123 125

CTTC[A/C]T[T/G]CAAATACTCTTTTCGAACAATTTCACCTGTTCCTTTCGTTGTCAGTG

207

CATCCTCTGTTTCTCCATCATCTCCTTCTACATC[T/A/C]GTTGCCCAATCTCAGGATCTC

241 242 254 271

AAGGTATCTAAT[T/A/C][G/A]CTTCCAAATAT[C/T]ACTATATAACGTGTAT[A/G]T

298 320

TTTTATGTGATTTAATGGAAAATTT[G/A]ATTTACTTGAATGCCAGATTA[A/T]ATCAGT

339

TCCTACCAAGCC[T/A]ATTGAAGGTCAAAAGACCGGTACCAGTGGTCTACGCAAAAAGGTG

408 409 410 411 413

CCTTTTCTTTTGGTTTTATTAAA[A/T][T/A][T/A][T/A]T[A/T]CACTTTGTTTGGA

440 467 468

TGATTGTTACCCG[C/T]TGTATTGTATTGTTAGTTCAAATACG[A/G][T/C]GTTTATTTTGATTGTTATATAAATTTGTTGTTTAGTATCATTTAAATTCATTTTCATTGTTAGCTACAAAAAGTCCCATTTTGTGTAAGGACCAATTTGGTGTGATTGCCTTGGTTACTTTTAATATTTTCTTCTCATTTATCACATTTTAATGTTTTATCCTTTACCCTATTTTTTCGTAGTAGCTACATCCCGTGCCTTACTTTTCTCGTAGGCTTATCAGACATATCGCAATGACGGAGAATGATACAATCTATCCAAATGTTGTATTTATTAAAATAATATTGTACGATACAATACAACACAATATGATACATAATGAAATAATACATAACAGCCCTCCAAGCAGAGTGAACTCCGCCTGCGTGAAGGTGTGCCAAAATAAATAGGAAAACCTCTCTACCACTTAAGGTAGGGTTAAGGCTGCGTATATTTTACCTTCCTCAGACCTCACTTGTGCGATTACACTGGTTTGTTATTGTTGTTGGTTTATTAAATTTTTTATTGCTTACTGTTCATTTGGATGACTTCTGCTTCTTTTGTCTATAGGTTAAAGTGTTTATGCAGGACAATTACCTTGCCAATTGGATAC

**Soluble starch synthase I, *SssI* (PGSC0003DMG402018552)**

Chr03:45608638..45609659

GGATACTCATGGGAAATAACAACTCCTGAAGGGGGATATGGGCTACATGAGCTGTTGAGCAGTAGACAGTCTGTTCTTAATGGTATGCGTGTTAGATGATTCCTTATGAGTGCTTTCTGATATTCTTCTTTTCCTCTTACTCGAATTGTAATTTGGTAGGATATATGTTGATTGACGAGGCTTATGTACCAGCATTAGCTTCATTTCTAGTCAAGTCCTAACTTTTGTTAAAACATGACCAGAGCCAAGGCTAATTGTATTAATGACCTTCACCATGCATTTCTGTTGTCTCTGAACTTTAGAAATTACTAGTTAATCTATCTCTACTTTTATGCAGGAATTACTAATGGAATAGATGTTAATGATTGGAACCCGTCGACAGATGAGCATATTGCTTCGCATTACTCCATCAATGACCTCTCCGGAAAGGTTATTAAACTTCTCAGTTTCACAATTTTGCAAATTAAGTGATAAAGTGCAGCTAGAGAAAAGGACTATTAGCATATTTGGCCAATCTTCCATAAATTGCTTATTTTGGGAAGTGTTTTTTATTAGAAGTACTTTTGGAGAATAGTAGTTTGTATTTGGCTAATCAATTTGAAAAAAAGCCTTTGTCAATATTAGAGCAGTAATTTGTGCTTGACAAAGGTTCCAATAGTGCTTCGAGGGGAAAAGCTATTTT

5803(indel A)

TTTCTTCCAACTTCAGCTACTACTCAAAAGTCAAAAGCAC|GCTTGGCTGAACAGACAATA

5824 5839 5850 5858

[C/T]GTCTACAAAAGCTT[C/A/T]TTAACTGCTG[C/T]GAAATTC[A/G]ATCCAAATG

5871 5877 5880 5906 5907

AGG[A/G]TTCCT[C/T]TA[G/A]AGGATTGTCCATAATCCATTAAAAG[G/A][G/C]AA

5913 5915 5932

CAG[T/C]C[C/T]AAAGGATATCATAGCA[T/C]TTACTTACTTTTCATGTTTATTCTACA

5964 5995 6001 6002

TGCA[G/A]TTGTGCACAAACTCTCTTTTTCTCTGTTCT[C/T]TACAT[A/G][C/A]CAC

6010 6015

TGCT[C/T]AAAT[T/A]GTCTCTGCTAACTTTCCATTTAATTATACAGGTTCAGTGCAAGACTGATCTGCAAAAGGAACTGGGCCTTCCAATTCGACCTGATTG

**Plastidial phosphoglucoisomerase 1, *PGI1-4* (PGSC0003DMG400012910)**

>chr04:64775817..64776308

Agcatctactcaccttcttcatctttcaaatctgaagtgaaatccattcacaaaatcacttcttcccaattgggttcaatttatttgcccaataaatcaagattccatgttcacgcggtggcgc

197(indel)

gtgaggtttcagcgagcttgtcggccggaaacaatgacgttgttcacaagtt|gaaggagaa

209 235 252

tg[t/a]tgggttggagaaaaacccgaatgct[c/t]tatggaagagatatgt[t/a]gact

267 270 277 297

ggctttatca[g/a]ca[c/t]aaggag[t/c]tgggtttgtatttggatat[t/c/a]agt

303 333 342

cg[a/g]gttggattcactgatgggtttcttgagga[a/g]atggagcc[t/c]cgtttgca

351 387

[a/g]aaggcgtttaaggatatggtggatttggagaaggg[a/g]gctattgcaaacccaga

435 438

tgagggtagaatggtgggtcactattggtt[g/a]ag[a/g]agtcctcatcttgctcccaactcatttctcaggttgcagattgagaatactcttgaagctgtttgccagtttgca

**L-type starch phosphorylase, chloroplastic/amyloplastic, *PHO1b* (PGSC0003DMG400028382)**

>Chr05:350160..351337

TGTTGCAAGAAAAGCTAAACCAAATGAGGATTCTGGATAATGTTGAAATACCAACTTCTGTTTTGGAGTTGCTTATAAAAGCCGAAGAAAATGCTGCTGATGTCGAAAAAGCAGCAGATGAAGAACAAGAAGAAGAAGGTAAGGATGACAGTAAAGATGAGGAAACTGAGGCTGTAAAGGCAGAAACTACGAACGAAGAGGAGGAAACTGAGGTTAAGAAGGTTGAGGTGGAGGATAGTCAAGCAAAAATAAAACGTATATTTGGGCCACATCCAAATAAATCACAGGTGGTTCACATGGCAAATCTATGTGTAGTTAGCGGGCATGCAGTTAACGGTGTTGCTGAGATTCATAGTGAAATAGTTAAAGATGAAGTTTTCAATGAATTTTACAAGGTACGATGATTATCACACGGCTAGAAGTGGCAGATATCA

3882

TTTTCAGTTCTCCTCTTTCAATGTTGTTGTTTTGATTGATCCAAACTCTTTGTTC[A/C]TC

3931 3934

GACAGTTATGGCCAGAGAAATTCCAAAACAAGACAAATGGTGTGAC[A/G]CC[A/T]AGAA

3958 3982 3990

GATGGCTAAGTTTCTGTAA[T/C]CCAGAGTTGAGTGAAATTATAAC[A/C]AAGTGGA[C/

4018 4038

T]AGGATCTGATGATTGGTTAGTAAACAC[T/A]GAAAAATTGGCAGAGCTTC[G/A]AAAG

4049 4052 4076 4088

GTAAAC[G/A]AG[T/C/G]TGTATCTGACTGTGATCTTATT[G/A]ATTCCTAAACC[G/A

4106 4109 4112 4122

]TCGTCAACAGCTTTCAT[A/G]AA[C/G/A]GG[A/G]TTATTTCCT[T/C]TAGTCCTTC

4181

TATGTGGAAAGAGATCTTTATAAACATCATAAGAACATGTGAAATGTAT[G/A]TACTAA[C

4188 4198 4206 4207

/T]TTCATGCAT[T/G]TAACTGT[T/C/G][G/T/A]AACAGTTTGCTGATAACGAAGAAC

4236 4248 4258

TCCA[G/A]TCTGAGTGGAG[G/A]AAGGCAAAA[G/A]GAAATAACAAAATGAAGATTGTC

4302 4319 4320

TCTCTCATTAAAGAAAAAAC[A/T]GGATACGTGGTCAGTC[C/G][C/T]GATGCAATGTT

4350 4356 4365

TGATGTTCAGATCAAGCG[C/T]ATCCA[T/C]GAGTATAA[A/G]AGGCAGCTATTAAATA

4383 4389 4404 4407 4413

T[A/T]TTTGG[A/T]ATCGTTTATCGCTA[T/C]AA[G/A]AAGAT[G/T]AAAGAAATGA

4431 4452 4457 4464

GCCCTGA[A/T]GAACGAAAAGAAAAGTTTGT[C/A]CCTC[G/A]AGTTTG[C/T]ATATT

4473 4496 4509

TGG[G/A]GGAAAAGCATTTGCTACATATG[T/C]TCAGGCCAAGAG[A/G]ATTGTAAAATTTATCACTGATGTAGGGGCAACAGTCAACCATGATCCCGAGATTGGTGATC

**Cycling DOF (DNA binding with one finger) factor 1, *StCDF1*, (PGSC0003DMG400018408)**

>chr05:4540349..4540939

Ccgcgatgtaatagcatggaaacaaagttctgttattacaacaattacaacgtcaaccagcctcgttacttctgcaagaactgccagagatattggactgctggagggacaatgagaaatgtgc

1458 1462 1475 ctgtgggatctgg[t/a]cgc[c/a]gaaagaacaaga[g/a]ttcttccatttcaaattat

1505

cctcttcaag[c/t]aggtcgggtcgaagcagcagctcacggaatgcatcttcctgcttt

1551 1572

[a/g]aggacaaatggaactgtcct[t/a]acatttggatcagataaacccctttgtgattc

1650

aatggtttctgcattgaacttagctgagaattcacataatatgaa[t/c]cgaaatgaattc

1671 1692

catggatc[c/t]gaacgaagaatgcctgcaat[c/t]gggaatgatcaatcaaatggaact

1770

tgtagtacagcctcaagtgtaactgacaaagaaagcagtgctggtactcatga[t/a]ttag

1776 1795 1812

c[a/t]aattggaataatttccag[c/a]catttcctcctcaagt[a/t]ccgtactttcag

1827 1875

gg[c/t]gctccgtggccttattctggctttccagtatcattctatccagcaac[a/t]ccg

1887

tactgggg[c/a]tgcaccgtagcaaacccttggaac

**Transcription factor, *StBEL5* (PGSC0003DMG400005930)**

Chr06:54712756..54713423

Cgattatggaagccaatggtagaagaaatgtacttggaagaagtgaagaatcaagaacaaaacagtactaatacttcaggagataacaaaaacaaagaggctccaaatgaagagaaacatccaattattactagcagcttattacaagatggtattactactactcaagcagaaatttctacctcaactatttcaacttcccctactgcaggtgcttcacttcatcatgctcacaatttctccttcct

2760 tggttcattcaacatggataatactactactactac[t/a]gttgatcatattgaaaacaa

2781 2784 2808 2814 2820

[c/a]gc[g/c]aaaaagcaaagaaatgacatgca[c/g/a]aagtt[t/c]tctcc[a/g]

2838 2853 2855

agtagtattctttcatc[t/c/g/a]gttgacatggaagc[c/t]a[a/g]agctagagaat

2874 2892

catcaaa[t/c]aaagggtttactaatcc[t/a]ttaatggcagcatacgcgatgggagatt

2952 2958 2960 2961

ttggaaggtttgatcctcatgatcaacaaat[c/g]accgc[g/a/c]a[a/g][t/c]ttt

3020

catggaaataatggtgtctctcttactttaggacttcctccttctgaaaacctag[c/t]ca

3032 3057 3062

tgccagtga[g/a]ccaacaaaattacctttctaatga[c/g]ttgg[g/c]aagtagg

3070

[c/t]ctgaaatggggagtcattacaatagaatgggatatgaaaacattgattttcagagtgggaataagcgatttcc

**Beta amylase 1, *BMY-8/2* (*BMY1*) (PGSC0003DMG400001855)**

>Chr08:50594548..50595107

gctactggagcatggtgacagaatagtagcagcaggagaaagtatataccaaggaactggggctaaactatctggaaaggtagctgggattcattggcattacaatactagatcacatgctgca

2505 2533

gagttaac[t/A]tcaggatattataatacaagacacaga[g/a]atggttatctacctata

2565 2576 2583

gcacgtatgttagc[g/a]aaacatggtg[c/t]tgtact[t/g]aactttacatgtatgga

2613 2625 2649

aatgagggatgg[t/g]gaacagcccca[g/a]agtgcaaactgttcaccagaagg[c/a]t

2657 2671 2686 2694

tagttc[a/g]acaagttaaaact[g/a]cagctagaactgct[a/g]aagtaga[a/g]ct

2751

tgctggagaaaatgctctagaaaggtatgatggaggagcattttctcaagtttt[g/a]gca

2767

acaagcatgtca[a/g]attctggaaatggattgagtgcatttacattcttgcgaatgaa

2811

[c/t]aaacggttgtttgagccagaaaattggcggaatctagtgcaatttgtgaagagcatgtctgaaggaggtcgaaatgctagccttccagagtgtgactcaagcaggacagacctctatgtaa

**Invertase, *INV-8/2* (PGSC0003DMG400004790)**

>Chr08:52703547..52704642

gttctcatcccaccacccggaattgggctcaaggacttccgtgaccccaccaccgcgtggaccacaccggaaggcaaatggcgtattactattggttcaaagattaataaaaccggaatctcat

2065 2075 2076

tggtctatgacacaattgattttaaaaaatttgaatt[g/A]ttgaagggt[g/A][c/T]g

2116

ttacatggtgtaccgggtacgggtatgtgggaatgtgt[g/A]gatttttacccggtttc[t

2134 2175 2182

/G]aaaattgttgaaaatgggcttgacacttcagaaaatgggc[c/A]tgcagt[g/A]aaa

2197

catgttttgaa[g/A]tccagtcttgacgatgatcgaaatgactattatgcccttggaactt

2255 2272

atgatgcaggg[g/A]caggaaaatgggtacc[t/A]gataatccaataattgatgttggta

2302 2320

ttgg[g/A]ttaagatatgattatgg[t/C]aatttttatgcatcaaaaacattttatgatc

2369 2371

aagaaaaaaagagaaga[g/A]t[t/A]ctttgggcttggattaaagaaactgatagtgaag

2425

ctgctgatatttgcaggg[T/C]ttgggcctcacttcaggtatctcttttttagcgtatcatttagattttgtccttatttttaaaagtcgtgcaagtagatacataatttgaagtgtgacaagtaatcaaaaatcagacaatgacagaacggcctatgattaagaactaaaaactaaaactttatacaacttttacaattcattgaacaaattggtaacgtgtgattttttattattttatttcgaaaacagtcaattccaagaactataaagtatgataagaagacaggaagcaacataattacatggccagtggctgaggtagagaatttgagatcaaacaacaatgaattcaacaaggttgtagttaaaccaggttcaattgtcccactagaagttggttctgccactcaggttagtacattttgtcgattaaattcaacttatatacattgacaatatatatacttatatcgttgaattattgttacagttggacataatggctgaatttgaaatagaccaaaatgtgttgaagaaagtagatggaagtaatgctacatatgattgtataaagagtggtggatctggtgaacg

**Phosphoglucan water dikinase, *PWD* (PGSC0003DMG400016613)**

>chr09:60571111..60571981

GGTCTGATGATCTATCTGATTGCAGTTTATAGTGACCAAGGTGCACCAGCATCATTTAACGT

10503 10543 10547

TCCTGC[A/T]GGAGCAGTTATTCCATTTGGTTCCATGGAAACGGCATTG[G/A]AAA[T/C

10576

]GAACAAGTTAATGGAGACCTTCACATTG[C/G]TTGTCGAACAGATAGAAACAGCTGAAAT

10629 10657

TGATGGCGGTGAACTTGATAAACA[T/C]TGTGAGGATCTCCAGAAGTTAATATCT[T/A]C

10689 10700 10701

TCTATTGCCTGGACAAGATGTCATTGAAAG[C/G]TTGGGAGAAG[T/C][A/G]TTTCCCG

10746 10758

GTAATGCACGTTTAATAGTGCGTTCAAGTGCTAATGT[C/T]GAGGACTTGGC[G/A]GG[G

10761 10770 10773

/A]ATGTCAGC[T/A]GC[T/C]GGACTTTATGATTCAATTCCTAATGTTAGCCCTTCAGAT

10815

CC[G/A]ATAAGGTTTGGACATGCTGTAGCCCGTGTTTGGGCCTCGTTGTATACTAGAAGAG

10911 10916 10917

CAGTACTGAGCCGCAGAGCTGCTGGTGTGTCCCAGAAAGA[C/T]GCTA[C/A][A/G]ATG

10923 10932 10962

GC[C/T]GTGCTAGT[G/T]CAAGAAATGCTTTCACCAGATTTATCTTT[C/T]GTCCTCCA

11022

CACACTGAGCCCAACGGACAACAATCATAACTTCATTGAGGCTGAAATTGC[A/T]CCTGGA

11064

CTCGGTGAAACACTCGCTTCAGGAACAAGGGGTAC[A/T]CCGTGGCGTCTATCTAGTGGTA

11112 11118

AATTTGACGACACAGTGCGCACACT[G/A]GCATT[C/T]GCCAACTTTAGTGAGGAGATG[

11140 11148 11151 11152

G/A]TTGTAGG[T/A]GG[C/T][A/C]ATTCCCCTGCTGACGGAGAAGTTATTCTCTTGACTGTCGATTATAGCAAGAAACCTTTAACAATCGACCCCATTTTCAGACGTCAGCTTGGTCAGAGGCTTGGCGCTGTTGGTTTCTACCTAGAACGCAAGTTTGGTTCTCCTCAAGATGTCG

**Invertase, apoplastic, *InvCD141* (PGSC0003DMG402028252)**

>chr10:55854799..55855817

Gg[c/t]ccaatgtattacaatggagtgtatcatttattctaccagtacaacccaaagggcg

276 280 288

caatatggggcaa[c/t]att[g/a]tttgggc[c/t]cattcggtctcaaaggacttgatc

339

aattggatcccacttgaacccgctat[c/t]tacccgtccaaagtatttgacaagtatggta

378

catggtc[c/t]gggtcagccacaatcttgccaggcaacaagcctgttattctctacac

426 440 444 462

[t/c]ggaattgtggatg[c/g]taa[c/t]aagacacaagtccaaaa[t/c]tatgcaatc

474 481 483

cc[g/a]gctaac[a/t]t[g/a]tctgatccatatcttcgtaagtggatcaagcccgataa

543

caatccattgattgttgctga[c/t]aaaaccatcaacaaaagccaatttcgtgatccaaca

582 585 601 621

ac[c/a]gc[t/a]tggatgggccgagat[g/a]gaaattggagaatcttggt[a/t]gg

624 630 667

[g/c]agtgt[g/a]aggaatcataggggaaaggttataatgtacaaaagt[g/a]ataag

673 701 714 717

[g/a]acttcatgaaatggaccaaagccaaac[a/t]cccactccactc[a/c/g]gc[c/t

720 765

]cc[g/c]ggtactggaaattgggaatgtcctgatttttttccagtgtcatt[g/a]aaaaa

775 798

taaa[a/g]atggtttggacacgtcatacaa[t/c]ggcaaagacattaaacatgttcttaa

843 862

ggttagctttgatgttac[t/a]aggtttgatcattacaca[a/g]ttggtacatatgacac

889 891 905

caaaaaggat[a/c]a[a/g]tactttccggata[a/g]cacttctattgatggatggaaag

939 980

gattgagact[t/c]gactatggtaactattacgcgtccaagacattctttgata[a/g]tg

1029 1030

gcaagaatcgtaggattttgttgggttgggctaatgaatcagatac[t/g][g/c]ttgata

1059

acgatgtgaggaaaggatgggc[c/t]ggagttcaccctattcctcgtaaaatttggcttga

1096 1135

t[c/t]ctagtggaaaacaattggttcaatggcctgttcaagaa[t/c]tagaaactctaagaaagaaaaaggtccaattaaataacaaaaagttgaacaagggagaaaaggttgaaatcaaaggaatcacagttgc

**Leucine aminopeptidase N*, LapN* (PGSC0003DMG400007831)**

>Chr12:2330749..2331596

GCTTCCTGGTCTTGGCTCAAAAAGGATTGCTCTAGTTGGGCTTGGCTCACCAACATCATCAACTGCTGCTTATCGCTGTTTAGGGGAGGCTGCTGCTGCAGCTGCCAAGTCTGCTCAGGCTAGT

2704 2707 2731 AATAT[C/T]GC[C/T]ATTGCTCTTGCTTCTACGGATGG[A/G]CTCTCTGCAGAATC

2746 2783 2790

[G/A]AAGCTTAGCTCTGCCTCTGCCATAACAACTGGTATC[C/G]ATTTTC[A/G]TG

2793 2799 2810 2813 2816 2831indel±A

[G/A**]**TCTTC[G/A]CTTAATTCAT[T/C]GA[A/G]CC[A/G]TATTGAGAAACTAA|CTTGGTTGATTTTCGTGAACATTGTAGGAGCTGTGCTGGGGACATTTGAAGATAATAGGTTTAAATCTGAGTCAAAGAAACCAACATTGAAATCTTTGGATATTCTTGGACTGGGGACTGGACCTGAGATAGAGAAGAAAATCAAGTATGCAGCAGATGTCTGTGCAGGTGTTATACTCGGAAGAGAGCTCGTCAATGCACCCGCCAATGTACTTACGCCTGGTTAGTGTTTTTCAATGCATTTCCTTGTT

3039 3097 3101 3107 3117

GTCCCTTTTA[T/A]TAG[T/A]ATG[A/C]CTATC[G/A]CCACACTAT[T/C]AAAATGC

3172

CGACTTTTCGCTGCAGCGGTACTTGCTGAAGAGGCCAAAAAGATTGC[G/A]TCCACTTATAGCGATGTCTTTTCTGCAAACATCTTGGATGTTGAGCAGTGCAAAGAATTGAAAATGGGATCCTATTTAGCAGTTGCTGCAGCTTCTGCAAATCCTGCTCATTTCATCCATTTGTCTTATAAGCCTAGTAGTGGAGAAATAAAAAAGAAGATAGCCTTGGTTGGAAAGGGATTAACTTTTGACAGGTAATTCTATCTTCTATAAGTTGGAAAAATAGAAATTTGATTTCTGACCTGGCTGC
