# Supplementary material for: Identification and reproducibility of diagnostic DNA markers for tuber starch and yield optimization in a novel association mapping population of potato (Solanum tuberosum L.)
Source: Theor Appl Genet. 2016 Jan 29;129:767–85. doi: 10.1007/s00122-016-2665-7 (PMC4799268; doi:10.1007/s00122-016-2665-7)
Supplement: Supplementary file 5 — Supplementary material 5 (DOCX 55 kb) [file 122_2016_2665_MOESM5_ESM.docx]

**Identification and reproducibility of diagnostic DNA markers for tuber starch and yield optimization in a novel association mapping population of potato (*Solanum tuberosum* L.)**

E. M. Schönhals, F. Ortega, L. Barandalla, A. Aragones, J. I. Ruiz de Galarreta, J.-C. Liao, R. Sanetomo, B. Walkemeier, E. Tacke, E. Ritter, C. Gebhardt

Theoretical and Applied Genetics

Corresponding author: Christiane Gebhardt, Max-Planck Institute for Plant Breeding Research, Cologne, Germany ([gebhardt@mpipz.mpg.de](mailto:gebhardt@mpipz.mpg.de)).

| **Online Resource 5**. Marker-trait associations in the QUEST(+LAN) population when applying the MM-PK model with p < 0.05 and including markers with MAF <1% (highlighted grey). Markers significant at p < 0.01 are highlighted light green. Markers significant at p < 0.001 are highlighted dark green.   \| Marker ^a^ \| Marker alleles ^b^ \| Position [Mbp] \| Allele frequency ^c^ \| TSC  p-value (R²) ^d^ \| TY  p-value (R²) ^d^ \| TSY  p-value (R²) ^d^ \| TW  p-value (R²) ^d^ \| TN  p-value (R²) ^d^ \| \| --- \| --- \| --- \| --- \| --- \| --- \| --- \| --- \| --- \| \| *STI043-a* \| 0/1 \| Chr01:03.50 \| 0.262 (1) \| 0.0262 (1.6) ↑ \| ns \| ns \| ns \| ns \| \| *STI043-c* \| 0/1 \|  \| 0.443 (1) \| 0.0154 (1.9) ↑ \| ns \| 0.0435 (1.4) ↑ \| ns \| ns \| \| *STI043-e* \| 0/1 \|  \| 0.496 (1) \| 0.0049 (2.6) ↑ \| ns \| 0.0054 (2.6) ↑ \| ns \| ns \| \| *CP12-2_snp71* \| C/T \| Chr01:62.72 \| 0.007 (C) \| ns \| ns \| 0.0298 (1.6) ↓ \| ns \| ns \| \| *CP12-2_snp84* \| G/A \|  \| 0.041 (A) \| ns \| 0.0196 (1.7) ↓ \| ns \| ns \| ns \| \| *CP12-2_snp142* \| G/A \|  \| 0.011 (A) \| 0.0416 (1.3) ↑ \| ns \| ns \| ns \| ns \| \| *CP12-2_snp327* \| T/C \|  \| 0.004 (C) \| ns \| ns \| ns \| ns \| 0.0002 (4.7) ↑ \| \| *STI009-h* \| 0/1 \| Chr01:84.89 \| 0.093 (1) \| ns \| ns \| ns \| ns \| 0.0086 (2.4) ↑ \| \| *STI009-i* \| 0/1 \|  \| 0.612 (1) \| 0.0343 (1.4) ↑ \| ns \| 0.0123 (2.1) ↑ \| ns \| ns \| \| *STI009-d* \| 0/1 \|  \| 0.018 (1) \| 0.0494 (1.2) ↑ \| ns \| ns \| ns \| ns \| \| *AGPaseS_snp1612* \| T/C \| Chr01:86.09 \| 0.090 (C) \| 0.0013 (3.4) ↓ \| ns \| ns \| ns \| ns \| \| *AGPaseS_snp1592* \| G/C \|  \| 0.228 (C) \| ns \| ns \| 0.0134 (2.1) \| ns \| 0.0234 (1.8) \| \| *AGPaseS_snp1511* \| A/G \|  \| 0.072 (G) \| 0.0339 (1.4) ↑ \| ns \| ns \| ns \| ns \| \| *AGPaseS_snp1457*  ^e^* \| G/C \|  \| 0.006 (C) \| 0.0358 (1.4) ↑ \| ns \| ns \| ns \| ns \| \| *AGPaseS_snp1411* ^e^* \| C/T \|  \| 0.006 (T) \| 0.0084 (2.2) ↑ \| ns \| ns \| ns \| ns \| \| *AGPaseS_snp1286* \| A/G \|  \| 0.246 (G) \| ns \| ns \| ns \| ns \| 0.0292 (1.6) \| \| *STM0038-c* \| 0/1 \| Chr02:19.62 \| 0.264 (1) \| ns \| ns \| ns \| 0.0122 (1.9) ↓ \| ns \| \| *STM0038-d* \| 0/1 \|  \| 0.493 (1) \| ns \| ns \| ns \| 0.0277 (1.5) ↑ \| ns \| \| *SSIV_snp2679* \| A/T \| Chr02:30.14 \| 0.062 (T) \| ns \| 0.0059 (2.4) ↑ \| 0.0498 (1.3) ↑ \| ns \| ns \| \| *SSIV_snp2719* \| A/T \|  \| 0.034 (A) \| 0.0270 (1.6) ↓ \| ns \| ns \| ns \| ns \| \| *STI024-d* \| 0/1 \| Chr02:44.53 \| 0.830 (1) \| ns \| ns \| ns \| 0.0356 (1.3) ↑ \| ns \| \| *STI024-c* \| 0/1 \|  \| 0.475 (1) \| 0.0218 (1.7) ↑ \| ns \| ns \| ns \| ns \| \| *Pain1-8c* \| 0/1 \| Chr03:39.25 \| 0.170 (1) \| 0.0002 (4.4) ↑ \| ns \| 0.0071 (2.5) ↑ \| ns \| ns \| \| *PGM1-3_snp468* \| T/C \| Chr03:41.50 \| 0.394 (C) \| ns \| 0.0387 (1.4) ↑ \| 0.0415 (1.4) ↑ \| ns \| ns \| \| *PGM1-3_snp467* \| A/G \|  \| 0.192 (G) \| ns \| 0.0450 (1.3) \| ns \| ns \| ns \| \| *PGM1-3_snp413 #* \| A/T \|  \| 0.035 (T) \| ns \| 0.0107 (2.1) ↓ \| 0.0078 (2.4) ↓ \| ns \| 0.0361 (1.5) ↓ \| \| *PGM1-3_snp411 #* \| T/A \|  \| 0.042 (A) \| ns \| 0.0233 (1.6) ↓ \| 0.0122 (2.1) ↓ \| ns \| ns \| \| *PGM1-3_snp410 ** \| T/A \|  \| 0.029 (A) \| ns \| ns \| 0.0390 (1.4) ↓ \| ns \| ns \| \| *PGM1-3_snp409 ** \| T/A \|  \| 0.029 (A) \| ns \| ns \| 0.0390 (1.4) ↓ \| ns \| ns \| \| *PGM1-3_snp408* \| A/T \|  \| 0.160 (T) \| ns \| ns \| 0.0452 (1.4) ↑ \| ns \| ns \| \| *PGM1-3_snp254 #* \| C/T \|  \| 0.031 (T) \| ns \| ns \| 0.0407 (1.4) ↓ \| ns \| ns \| \| *SssI_snp5850* \| C/T \| Chr03:45.89 \| 0.094 (T) \| 0.0122 (2.0) ↑ \| ns \| ns \| 0.0493 (1.2) \| ns \| \| *SssI_snp5858* \| A/G \|  \| 0.283 (G) \| ns \| ns \| ns \| 0.0495 (1.2) \| ns \| \| *SssI_snp5871* \| A/G \|  \| 0.446 (G) \| ns \| ns \| ns \| 0.0085 (2.1) ↓ \| ns \| \| *SssI_snp5877** \| C/T \|  \| 0.116 (T) \| 0.0198 (1.7) ↑ \| ns \| ns \| 0.0295 (1.4) ↓ \| ns \| \| *SssI_snp5880** \| G/A \|  \| 0.113 (A) \| 0.0329 (1.5) ↑ \| ns \| ns \| 0.0414 (1.3) ↓ \| ns \| \| *SssI_snp5907* \| G/C \|  \| 0.046 (C) \| ns \| ns \| ns \| 0.0025 (2.8) ↑ \| 0.0108 (2.2) ↑ \| \| *SssI_snp5995* \| C/T \|  \| 0.008 (T) \| ns \| ns \| ns \|  \| 0.0053 (2.7) ↑ \| \| *SssI_snp6010* \| C/T \|  \| 0.055 (T) \| ns \| ns \| ns \| 0.0242 (1.5) ↓ \| ns \| \| *SssI_snp6015 ^e^* \| T/A \|  \| 0.007 (A) \| ns \| 0.0045 (2.6) ↑ \| 0.0170 (1.9) ↑ \| 0.0031 (2.7) ↑ \| ns \| \| *M17-b* \| 0/1 \| Chr03:49.71 \| 0.423 (1) \| 0.0029 (2.9) ↑ \| ns \| 0.0142 (2.0) ↑ \| ns \| 0.0233 (1.8) ↑ \| \| *M4-a* \| 0/1 \|  \| 0.979 (1) \| 0.0031 (2.8) ↑ \| ns \| 0.0181 (1.9) ↑ \| ns \| 0.0095 (2.3) ↑ \| \| *STM3016-f* \| 0/1 \| Chr04:06.60 \| 0.297 (1) \| 0.0035 (2.8) ↑ \| ns \| ns \| 0.0425 (1.2) \|  \| \| *STM3016-d* \| 0/1 \|  \| 0.613 (1) \| ns \| ns \| ns \| ns \| 0.0099 (2.3) ↑ \| \| *PGI1-4_snp333* \| A/G \| Chr04:64.78 \| 0.035 (G) \| 0.0073 (2.3) ↑ \| ns \| ns \| ns \| ns \| \| *PGI1-4_snp303** \| A/G \|  \| 0.087 (G) \| 0.0196 (1.7) ↓ \| ns \| ns \| ns \| ns \| \| *PGI1-4_snp267** \| G/A \|  \| 0.082 (A) \| 0.0078 (2.3) ↓ \| ns \| ns \| ns \| ns \| \| *PGI1-4_snp252** \| T/A \|  \| 0.082 (A) \| 0.0060 (2.4) ↓ \| ns \| ns \| ns \| ns \| \| *PGI1-4_snp235** \| C/T \|  \| 0.108 (T) \| 0.0117 (2.0) ↓ \| ns \| ns \| ns \| ns \| \| *PGI1-4_indel202* \| 0/1 \|  \| 0.305 (1) \| 0.0099 (2.1) ↓ \| ns \| ns \| ns \| ns \| \| *STI001-b ^e^* \| 0/1 \| Chr04:68.72 \| 0.404 (1) \| 0.0293 (1.5) \| ns \| ns \| 0.0035 (2.6) ↓ \| ns \| \| *STI020-d* \| 0/1 \| Chr04:70.15 \| 0.555 (1) \| 0.0490 (1.2) ↑ \| ns \| 0.0147 (2.0) ↑ \| ns \| ns \| \| *STI020-c* \| 0/1 \|  \| 0.292 (1) \| ns \| 0.0241 (1.6) \| 0.0214 (1.8) ↑ \| ns \| 0.0059 (2.6) ↑ \| \| *PHO1b_indel4163* \| 0/1 \| Chr05:00.35 \| 0.026 (1) \| ns \| ns \| 0.0455 (1.4) ↑ \| ns \| ns \| \| *PHO1b _snp4188* \| C/T \|  \| 0.012 (T) \| 0.0435 (1.3) ↓ \| ns \| ns \| ns \| ns \| \| *PHO1b_snp4198* \| T/G \|  \| 0.070 (G) \| ns \| ns \| ns \| ns \| 0.0282 (1.7) \| \| *PHO1b _snp4319* \| C/G \|  \| 0.310 (C) \| ns \| 0.0142 (1.9) ↑ \| 0.0088 (2.3) ↑ \| 0.0160 (1.8) ↑ \| ns \| \| *PHO1b_snp4431 ^f^* \| A/T \|  \| 0.006 (T) \| ns \| ns \| ns \| ns \| 0.0077 (2.5) ↓ \| \| *PHO1b_snp4452* \| C/A \|  \| 0.006 (A) \| ns \| ns \| ns \| ns \| 0.0162 (2.0) \| \| *STI058-b* \| 0/1 \| Chr05:4.17 \| 0.409 (1) \| ns \| ns \| 0.0106 (2.2) \| 0.0018 (3.0) ↑ \| ns \| \| *STI058-e* \| 0/1 \|  \| 0.069 (1) \| ns \| 0.0367 (1.4) ↑ \| 0.0353 (1.5) ↑ \| ns \| ns \| \| *STI058-a* \| 0/1 \|  \| 0.468 (1) \| ns \| 0.0170 (1.8) \| 0.0425 (1.4) \| ns \| ns \| \| *STI058-g* \| 0/1 \|  \| 0.029 (1) \| ns \| ns \| ns \| ns \| 0.0008 (3.9) ↑ \| \| *StCDF1_snp1458 ^§^* \| T/A \| Chr05:04.54 \| 0.071 (A) \| 0.0191 (1.8) ↑ \| ns \| 0.0186 (1.9) ↑ \| ns \| 0.0363 (1.5) \| \| *StCDF1_snp1462 ^§^* \| C/A \|  \| 0.064 (A) \| ns \| ns \| 0.0228 (1.8) ↑ \| ns \| 0.0297 (1.6) \| \| *StCDF1_snp1505* \| C/T \|  \| 0.015 (T) \| 0.0254 (1.6) ↓ \| ns \| ns \| ns \| ns \| \| *StCDF1_snp1551** \| A/G \|  \| 0.193 (G) \| ns \| ns \| 0.0048 (2.7) ↑ \| 0.0167 (1.7) ↑ \| ns \| \| *StCDF1_snp1692** \| C/T \|  \| 0.194 (T) \| ns \| 0.0118 (2.0) ↑ \| 0.0106 (2.2) ↑ \| 0.0202 (1.6) ↑ \| ns \| \| *StCDF1_snp1770 #* \| T/A \|  \| 0.196 (A) \| ns \| ns \| ns \| 0.0123 (1.9) \| ns \| \| *StCDF1_snp1776 #* \| A/T \|  \| 0.202 (T) \| ns \| ns \| ns \| 0.0117 (1.9) \| ns \| \| *StCDF1_snp1812* \| A/T \|  \| 0.151 (T) \| ns \| 7.38E-05 (5.1) ↑ \| 6.24E-05 (5.6) ↑ \| 0.0097 (2.0) ↑ \| ns \| \| *StCDF1_snp1827 §* \| C/T \|  \| 0.130 (T) \| ns \| ns \| ns \| 0.0158 (1.8) \| ns \| \| *StCDF1_snp1875 §* \| A/T \|  \| 0.131 (T) \| ns \| ns \| ns \| 0.0327 (1.4) \| ns \| \| *StCDF1_snp1887* \| C/A \|  \| 0.083 (A) \| ns \| ns \| ns \| ns \| 0.0119 (2.2) \| \| *STG0021-e* \| 0/1 \| Chr05:45.58 \| 0.587 (1) \| 0.0349 (1.4) \| ns \| ns \| ns \| ns \| \| *STG0021-a* \| 0/1 \|  \| 0.039 (1) \| ns \| ns \| ns \| ns \| 0.0012 (3.6) ↑ \| \| *StBEL5_snp3020* \| C/T \| Chr06:54.71 \| 0.043 (T) \| ns \| 0.0324 (1.5) ↓ \| ns \| 0.0372 (1.3) ↓ \| ns \| \| *StBEL5_snp2960 ^e^* \| A/G \|  \| 0.003 (G) \| ns \| ns \| ns \| ns \| 5.07E-06 (7.4) ↑ \| \| *StBEL5_snp2820* \| A/G \|  \| 0.007 (G) \| ns \| ns \| ns \| ns \| 0.0102 (2.3) ↑ \| \| *StBEL5_snp2781* \| C/A \|  \| 0.004 (A) \| 0.0252 (1.6) ↑ \| ns \| ns \| ns \| 0.0335 (1.6) ↑ \| \| *StBEL5_snp2760* \| T/A \|  \| 0.013 (A) \| ns \| ns \| 0.0431 (1.4) ↓ \| ns \| ns \| \| *STI004-f* \| 0/1 \| Chr06:55.86 \| 0.381 (1) \| ns \| ns \| 0.0415 (1.4) ↓ \| ns \| ns \| \| *STI004-d* \| 0/1 \|  \| 0.214 (1) \| ns \| 0.0372 (1.4) ↑ \| 0.0497 (1.3) ↑ \| ns \| 0.0015 (3.5) ↑ \| \| *STI004-k ^e^* \| 0/1 \|  \| 0.160 (1) \| ns \| ns \| ns \| ns \| 0.0054 (2.7) ↑ \| \| *STM1043-d(Sus-7/2) ^e^* \| 0/1 \| Chr07:40.64 \| 0.011 (1) \| ns \| 0.0432 (1.3) ↑ \| 0.0229 (1.8) ↑ \| ns \| 9.21E-06 (7.0) ↑ \| \| *STM3009-b* \| 0/1 \| Chr07:56.50 \| 0.007 (1) \| 0.0019 (3.1) ↑ \| ns \| ns \| ns \| ns \| \| *SSR327-a* \| 0/1 \| Chr08:35.52 \| 0.057 (1) \| 0.0316 (1.5) \| ns \| ns \| ns \| ns \| \| *BMY-8/2_snp2565* \| G/A \| Chr08:50.59 \| 0.030 (A) \| 0.0411 (1.3) \| ns \| ns \| ns \| ns \| \| *BMY-8/2_snp2583* \| T/G \|  \| 0.119 (G) \| ns \| ns \| ns \| 0.0274 (1.5) ↓ \| ns \| \| *BMY-8/2_snp2671* \| G/A \|  \| 0.177 (A) \| ns \| ns \| ns \| 0.0191 (1.7) ↑ \| ns \| \| *INV-8/2_snp2134* \| T/G \| Chr08:52.70 \| 0.3922 (G) \| ns \| ns \| ns \| ns \| 0.0201 (1.9) \| \| *STM1104-f(GBSS I)* \| 0/1 \| Chr08:56.78 \| 0.125 (1) \| 0.0019 (3.1) ↑ \| ns \| ns \| ns \| ns \| \| *InvGE-6f ^e^* \| 0/1 \| Chr09:02.47 \| 0.291 (1) \| ns \| ns \| ns \| 0.0053 (2.4) ↑ \| ns \| \| *STM1052-b(InvGE/GF)* \| 0/1 \| Chr09:02.47 \| 0.327 (1) \| ns \| ns \| 0.0243 (1.7) \| 0.0194 (1.7) \| ns \| \| *STM1052-c(InvGE/GF)* \| 0/1 \|  \| 0.712 (1) \| ns \| 0.0048 (2.5) ↑ \| 0.0021 (3.2) ↑ \| ns \| ns \| \| *STM3012-b* \| 0/1 \| Chr09:03.96 \| 0.149 (1) \| ns \| ns \| ns \| 0.0381 (1.3) \| ns \| \| *STM3012-a* \| 0/1 \|  \| 0.473 (1) \| 0.0206 (1.7) \| ns \| ns \| ns \| ns \| \| *STI014-a* \| 0/1 \| Chr09:55.35 \| 0.274 (1) \| ns \| ns \| ns \| 0.0218 (1.6) ↑ \| ns \| \| *STI014-c* \| 0/1 \|  \| 0.456 (1) \| ns \| ns \| ns \| 0.0185 (1.7) ↑ \| ns \| \| *STI014-d* \| 0/1 \|  \| 0.441 (1) \| ns \| ns \| ns \| 0.0344 (1.4) ↑ \| ns \| \| *PWD_snp10547* * \| T/C \| Chr09:60.56 \| 0.371 (T) \| ns \| ns \| ns \| ns \| 0.0036 (2.9) ↑ \| \| *PWD_snp10657* # \| T/A \|  \| 0.037 (A) \| 0.0043 (2.6) ↓ \| ns \| ns \| ns \| ns \| \| *PWD_snp10689* \| C/G \|  \| 0.142 (G) \| 0.0387 (1.4) \| ns \| ns \| ns \| ns \| \| *PWD_snp10700* # \| T/C \|  \| 0.040 (C) \| 0.0114 (2.1) ↓ \| ns \| ns \| ns \| ns \| \| *PWD_snp10701* \| A/G \|  \| 0.131 (G) \| 0.0476 (1.3) \| ns \| ns \| ns \| ns \| \| *PWD_snp10746* \| C/T \|  \| 0.015 (T) \| 0.0471 (1.3) \| ns \| ns \| ns \| ns \| \| *PWD_snp10758* \| G/A \|  \| 0.017 (A) \| ns \| ns \| ns \| 0.0320 (1.4) \| ns \| \| *PWD_snp10761* # \| G/A \|  \| 0.037 (A) \| 0.0043 (2.6) ↓ \| ns \| ns \| ns \| ns \| \| *PWD_snp10911 ** \| C/T \|  \| 0.367 (C) \| ns \| ns \| ns \| ns \| 0.0034 (3.0) ↑ \| \| *PWD_snp10916* “ \| C/A \|  \| 0.293 (A) \| 0.0017 (3.2) ↓ \| ns \| ns \| ns \|  \| \| *PWD_snp10917 “* \| A/G \|  \| 0.304 (G) \| 0.0464 (1.3) ↓ \| ns \| ns \| ns \| 0.0121 (2.2) ↑ \| \| *PWD_snp10923 “* \| C/T \|  \| 0.296 (T) \| 0.0017 (3.2) ↓ \| ns \| ns \| ns \| ns \| \| *PWD_snp10932* \| G/T \|  \| 0.012 (T) \| 0.0242 (1.6) ↑ \| ns \| ns \| ns \| ns \| \| *PWD_snp10962 ** \| C/T \|  \| 0.364 (C) \| ns \| ns \| ns \| ns \| 0.0040 (2.9) ↑ \| \| *STM2012-c* \| 0/1 \| Chr10:01.12 \| 0.057 (1) \| ns \| 0.0223 (1.7) ↓ \| ns \| ns \| ns \| \| *STG0025-d ^e^* \| 0/1 \| Chr10:33.54 \| 0.021 (1) \| ns \| 0.0410 (1.3) ↑ \| 0.0057 (2.6) ↑ \| ns \| ns \| \| *STG0025-c* \| 0/1 \|  \| 0.801 (1) \| ns \| ns \| 0.0477 (1.3) ↑ \| ns \| ns \| \| *Rca-1a* \| 0/1 \| Chr10:50.94 \| 0.443 (1) \| 0.0076 (2.3) ↓ \| ns \| ns \| ns \| ns \| \| *STM1106-g(InvCD141)* \| 0/1 \| Chr10:55.85 \| 0.647 (1) \| ns \| ns \| ns \| 0.0480 (1.2) ↑ \| ns \| \| *STM1106-b(InvCD141)* \| 0/1 \|  \| 0.122 (1) \| 0.0007 (3.7) ↓ \| ns \| 0.0317 (1.6) ↓ \| ns \| ns \| \| *InvCD141-Sa** \| 0/1 \| Chr10:55.85 \| 0.665 (1) \| 2.57E-05 (5.8) ↓ \| ns \| ns \| ns \| ns \| \| *InvCD141_snp280** \| G/A \| Chr10:55.85 \| 0.129 (A) \| 1.29E-06 (7.8) ↓ \| ns \| ns \| ns \| ns \| \| *InvCD141_snp288** \| C/T \|  \| 0.166 (T) \| 1.29E-06 (6.3) ↓ \| ns \| 0.0281 (1.6) \| ns \| ns \| \| *InvCD141_snp339** \| C/T \|  \| 0.142 (T) \| 1.22E-06 (7.8) ↓ \| ns \| ns \| ns \| ns \| \| *InvCD141_snp378 #* \| C/T \|  \| 0.204 (T) \| 0.0034 (2.8) ↑ \| ns \| ns \| ns \| ns \| \| *InvCD141_snp426* \| T/C \|  \| 0.200 (C) \| 1.18E-06 (9.4) ↓ \| ns \| ns \| ns \| ns \| \| *InvCD141_snp440* \| C/G \|  \| 0.221 (G) \| 0.0417 (1.3) \| ns \| ns \| ns \| ns \| \| *InvCD141_snp462 #* \| T/C \|  \| 0.434 (T) \| 0.0050 (2.5) ↑ \| ns \| ns \| ns \| ns \| \| *InvCD141_snp474* \| G/A \|  \| 0.298 (A) \| ns \| ns \| ns \| ns \| 0.0442 (1.4) \| \| *InvCD141_snp481 ^e^* \| A/T \|  \| 0.006 (T) \| ns \| 0.0096 (2.1) ↑ \| 0.0052 (2.7) ↑ \| ns \| 0.0001 (5.2) ↑ \| \| *InvCD141_snp543** \| C/T \|  \| 0.140 (T) \| 5.77E-07 (8.3) ↓ \| ns \| 0.0206 (1.8) \| ns \| 0.0142 (2.1) \| \| *InvCD141_snp582* \| C/A \|  \| 0.399 (A) \| 0.0117 (2.0) \| ns \| ns \| ns \| ns \| \| *InvCD141_snp624 #* \| G/C \|  \| 0.192 (C) \| 0.0444 (1.3) ↑ \| ns \| ns \| ns \| ns \| \| *InvCD141_snp630** \| G/A \|  \| 0.130 (A) \| 5.02E-05 (5.4) ↓ \| ns \| ns \| ns \| ns \| \| *STM0037-k* \| 0/1 \| Chr11:08.21 \| 0.007 (1) \| 0.0218 (1.7) ↓ \| ns \| ns \| ns \| ns \| \| *STM0037-e* \| 0/1 \|  \| 0.270 (1) \| 0.0403 (1.3) \| ns \| 0.0354 (1.5) \| 0.0435 (1.2) ↑ \| ns \| \| *STM0037-g* \| 0/1 \|  \| 0.814 (1) \| 0.0270 (1.6) ↓ \| ns \| 0.0141 (2.0) ↓ \| ns \| ns \| \| *STI028-f* \| 0/1 \| Chr11:37.97 \| 0.317 (1) \| ns \| ns \| ns \| 0.0006 (3.6) ↑ \| ns \| \| *STI028-a* \| 0/1 \|  \| 0.427 (1) \| ns \| 0.0187 (1.8) \| ns \| ns \| ns \| \| *STI028-b ^e^* \| 0/1 \|  \| 0.011 (1) \| 0.0016 (3.2) ↑ \| ns \| ns \| ns \| ns \| \| *LapN_snp2746* \| G/A \| Chr12:02.34 \| 0.091 (A) \| 1.56E-05 (6.1) ↑ \| 0.0237 (1.6) ↑ \| 0.0006 (4.1) ↑ \| ns \| 0.0017 (3.4) ↑ \| \| *LapN_snp2783* \| C/G \|  \| 0.020 (G) \| ns \| ns \| ns \| 0.0123 (1.9) ↓ \| 0.0056 (2.7) ↑ \| \| *LapN_snp2813* \| A/G \|  \| 0.037 (G) \| ns \| ns \| 0.0419 (1.4) \| ns \| ns \| \| *LapN_indel2831* \| 0/1 \|  \| 0.858 (1) \| 0.0459 (1.3) ↑ \| ns \| ns \| ns \| ns \| \| *LapN_snp3097* \| T/A \|  \| 0.491 (T) \| 0.0401 (1.3) ↑ \| ns \| ns \| ns \| ns \| \| *LapN_snp3117* \| T/C \|  \| 0.180 (C) \| 0.0004 (4.1) ↑ \| ns \| 0.0453 (1.4) ↑ \| ns \| ns \| \| *SSR20-x* \| 0/1 \| Chr12:11.26 \| 0.989 (1) \| ns \| ns \| ns \| ns \| 0.0148 (2.0) ↑ \| \| *SSR20-b* \| 0/1 \|  \| 0.117 (1) \| ns \| 0.0214 (1.7) \| ns \| 0.0340 (1.4) \| ns \| \| *SSR20-h* \| 0/1 \|  \| 0.121 (1) \| ns \| 0.0117 (2.0) \| ns \| 0.0478 (1.2) \| ns \| \| *SSR20-c* \| 0/1 \|  \| 0.656 (1) \| ns \| 0.0167 (1.8) \| 0.0038 (2.9) ↓ \| ns \| ns \| \| *STM0030-h* \| 0/1 \| Chr12:22.81 \| 0.021 (1) \| 0.0203 (1.7) ↓ \| ns \|  \| ns \| ns \| \| *STM0030-e* \| 0/1 \|  \| 0.507 (1) \| ns \| 0.0035 (2.7) ↑ \| 0.0033 (2.9) ↑ \| ns \| 0.0262 (1.7) ↑ \| \| *STM0030-f* \| 0/1 \|  \| 0.007 (1) \| ns \| ns \| ns \| 0.04185 (1.3) ↓ \| ns \| \| *STM0030-g* \| 0/1 \|  \| 0.624 (1) \| 0.0347 (1.4) ↓ \| ns \| ns \| ns \| ns \| \| *STM0003-a* \| 0/1 \| Chr12:60.05 \| 0.699 (1) \| ns \| ns \| 0.0225 (1.8) ↑ \| ns \| ns \| \| *STM0003-d* \| 0/1 \|  \| 0.440 (1) \| ns \| ns \| ns \| 0.0061 (2.3) ↓ \| 0.0061 (2.6) ↑ \|   ^a^ The same symbol next to SNP markers in the same gene indicates that the SNPs are in very strong LD (Supplemental Table S4) and form a haplotype;  ^b^ The nucleotide present in the reference potato genome sequence (*S. phureja*) is on the left position, the alternative allele (*S. tuberosum*) on the right;  ^c^ For SNPs the minor allele frequency (MAF) is shown with the minor frequency SNP allele in parenthesis; MAF was calculated including the allele dosage. The ‘allele’ frequency of microsatellite and allele specific PCR markers corresponds to the frequency of the presence (1) of the marker allele without counting allele dosage.  ^d^ ns = not significant at α = 0.05; Arrows indicate the direction of the effect of presence of the microsatellite or PCR marker allele or the effect of increasing dosage of the minor frequency SNP allele on the trait: ↑ increasing, **↓** decreasing mean values for TSC, TY, TSY, TW and TN.  *^e^* The minor frequency SNP/SSR allele was absent in landraces  *^f^* The minor frequency SNP allele was specific for landraces |  |  |  |  |  |
| --- | --- | --- | --- | --- | --- | --- | --- | --- | --- | --- | --- | --- | --- | --- | --- | --- | --- | --- | --- | --- | --- | --- | --- | --- | --- | --- | --- | --- | --- | --- | --- | --- | --- | --- | --- | --- | --- | --- | --- | --- | --- | --- | --- | --- | --- | --- | --- | --- | --- | --- | --- | --- | --- | --- | --- | --- | --- | --- | --- | --- | --- | --- | --- | --- | --- | --- | --- | --- | --- | --- | --- | --- | --- | --- | --- | --- | --- | --- | --- | --- | --- | --- | --- | --- | --- | --- | --- | --- | --- | --- | --- | --- | --- | --- | --- | --- | --- | --- | --- | --- | --- | --- | --- | --- | --- | --- | --- | --- | --- | --- | --- | --- | --- | --- | --- | --- | --- | --- | --- | --- | --- | --- | --- | --- | --- | --- | --- | --- | --- | --- | --- | --- | --- | --- | --- | --- | --- | --- | --- | --- | --- | --- | --- | --- | --- | --- | --- | --- | --- | --- | --- | --- | --- | --- | --- | --- | --- | --- | --- | --- | --- | --- | --- | --- | --- | --- | --- | --- | --- | --- | --- | --- | --- | --- | --- | --- | --- | --- | --- | --- | --- | --- | --- | --- | --- | --- | --- | --- | --- | --- | --- | --- | --- | --- | --- | --- | --- | --- | --- | --- | --- | --- | --- | --- | --- | --- | --- | --- | --- | --- | --- | --- | --- | --- | --- | --- | --- | --- | --- | --- | --- | --- | --- | --- | --- | --- | --- | --- | --- | --- | --- | --- | --- | --- | --- | --- | --- | --- | --- | --- | --- | --- | --- | --- | --- | --- | --- | --- | --- | --- | --- | --- | --- | --- | --- | --- | --- | --- | --- | --- | --- | --- | --- | --- | --- | --- | --- | --- | --- | --- | --- | --- | --- | --- | --- | --- | --- | --- | --- | --- | --- | --- | --- | --- | --- | --- | --- | --- | --- | --- | --- | --- | --- | --- | --- | --- | --- | --- | --- | --- | --- | --- | --- | --- | --- | --- | --- | --- | --- | --- | --- | --- | --- | --- | --- | --- | --- | --- | --- | --- | --- | --- | --- | --- | --- | --- | --- | --- | --- | --- | --- | --- | --- | --- | --- | --- | --- | --- | --- | --- | --- | --- | --- | --- | --- | --- | --- | --- | --- | --- | --- | --- | --- | --- | --- | --- | --- | --- | --- | --- | --- | --- | --- | --- | --- | --- | --- | --- | --- | --- | --- | --- | --- | --- | --- | --- | --- | --- | --- | --- | --- | --- | --- | --- | --- | --- | --- | --- | --- | --- | --- | --- | --- | --- | --- | --- | --- | --- | --- | --- | --- | --- | --- | --- | --- | --- | --- | --- | --- | --- | --- | --- | --- | --- | --- | --- | --- | --- | --- | --- | --- | --- | --- | --- | --- | --- | --- | --- | --- | --- | --- | --- | --- | --- | --- | --- | --- | --- | --- | --- | --- | --- | --- | --- | --- | --- | --- | --- | --- | --- | --- | --- | --- | --- | --- | --- | --- | --- | --- | --- | --- | --- | --- | --- | --- | --- | --- | --- | --- | --- | --- | --- | --- | --- | --- | --- | --- | --- | --- | --- | --- | --- | --- | --- | --- | --- | --- | --- | --- | --- | --- | --- | --- | --- | --- | --- | --- | --- | --- | --- | --- | --- | --- | --- | --- | --- | --- | --- | --- | --- | --- | --- | --- | --- | --- | --- | --- | --- | --- | --- | --- | --- | --- | --- | --- | --- | --- | --- | --- | --- | --- | --- | --- | --- | --- | --- | --- | --- | --- | --- | --- | --- | --- | --- | --- | --- | --- | --- | --- | --- | --- | --- | --- | --- | --- | --- | --- | --- | --- | --- | --- | --- | --- | --- | --- | --- | --- | --- | --- | --- | --- | --- | --- | --- | --- | --- | --- | --- | --- | --- | --- | --- | --- | --- | --- | --- | --- | --- | --- | --- | --- | --- | --- | --- | --- | --- | --- | --- | --- | --- | --- | --- | --- | --- | --- | --- | --- | --- | --- | --- | --- | --- | --- | --- | --- | --- | --- | --- | --- | --- | --- | --- | --- | --- | --- | --- | --- | --- | --- | --- | --- | --- | --- | --- | --- | --- | --- | --- | --- | --- | --- | --- | --- | --- | --- | --- | --- | --- | --- | --- | --- | --- | --- | --- | --- | --- | --- | --- | --- | --- | --- | --- | --- | --- | --- | --- | --- | --- | --- | --- | --- | --- | --- | --- | --- | --- | --- | --- | --- | --- | --- | --- | --- | --- | --- | --- | --- | --- | --- | --- | --- | --- | --- | --- | --- | --- | --- | --- | --- | --- | --- | --- | --- | --- | --- | --- | --- | --- | --- | --- | --- | --- | --- | --- | --- | --- | --- | --- | --- | --- | --- | --- | --- | --- | --- | --- | --- | --- | --- | --- | --- | --- | --- | --- | --- | --- | --- | --- | --- | --- | --- | --- | --- | --- | --- | --- | --- | --- | --- | --- | --- | --- | --- | --- | --- | --- | --- | --- | --- | --- | --- | --- | --- | --- | --- | --- | --- | --- | --- | --- | --- | --- | --- | --- | --- | --- | --- | --- | --- | --- | --- | --- | --- | --- | --- | --- | --- | --- | --- | --- | --- | --- | --- | --- | --- | --- | --- | --- | --- | --- | --- | --- | --- | --- | --- | --- | --- | --- | --- | --- | --- | --- | --- | --- | --- | --- | --- | --- | --- | --- | --- | --- | --- | --- | --- | --- | --- | --- | --- | --- | --- | --- | --- | --- | --- | --- | --- | --- | --- | --- | --- | --- | --- | --- | --- | --- | --- | --- | --- | --- | --- | --- | --- | --- | --- | --- | --- | --- | --- | --- | --- | --- | --- | --- | --- | --- | --- | --- | --- | --- | --- | --- | --- | --- | --- | --- | --- | --- | --- | --- | --- | --- | --- | --- | --- | --- | --- | --- | --- | --- | --- | --- | --- | --- | --- | --- | --- | --- | --- | --- | --- | --- | --- | --- | --- | --- | --- | --- | --- | --- | --- | --- | --- | --- | --- | --- | --- | --- | --- | --- | --- | --- | --- | --- | --- | --- | --- | --- | --- | --- | --- | --- | --- | --- | --- | --- | --- | --- | --- | --- | --- | --- | --- | --- | --- | --- | --- | --- | --- | --- | --- | --- | --- | --- | --- | --- | --- | --- | --- | --- | --- | --- | --- | --- | --- | --- | --- | --- | --- | --- | --- | --- | --- | --- | --- | --- | --- | --- | --- | --- | --- | --- | --- | --- | --- | --- | --- | --- | --- | --- | --- | --- | --- | --- | --- | --- | --- | --- | --- | --- | --- | --- | --- | --- | --- | --- | --- | --- | --- | --- | --- | --- | --- | --- | --- | --- | --- | --- | --- | --- | --- | --- | --- | --- | --- | --- | --- | --- | --- | --- | --- | --- | --- | --- | --- | --- | --- | --- | --- | --- | --- | --- | --- | --- | --- | --- | --- | --- | --- | --- | --- | --- | --- | --- | --- | --- | --- | --- | --- | --- | --- | --- | --- | --- | --- | --- | --- | --- | --- | --- | --- | --- | --- | --- | --- | --- | --- | --- | --- | --- | --- | --- | --- | --- | --- | --- | --- | --- | --- | --- | --- | --- | --- | --- | --- | --- | --- | --- | --- | --- | --- | --- | --- | --- | --- | --- | --- | --- | --- | --- | --- | --- | --- | --- | --- | --- | --- | --- | --- | --- | --- | --- | --- | --- | --- | --- | --- | --- | --- | --- | --- | --- | --- | --- | --- | --- | --- | --- | --- | --- | --- | --- | --- | --- | --- | --- | --- | --- | --- | --- | --- | --- | --- | --- | --- | --- | --- | --- | --- | --- | --- | --- | --- | --- | --- | --- | --- | --- | --- | --- | --- | --- | --- | --- | --- | --- | --- | --- | --- | --- | --- | --- | --- | --- | --- | --- | --- | --- | --- | --- | --- | --- | --- | --- | --- | --- | --- | --- | --- | --- | --- | --- | --- | --- | --- | --- | --- | --- | --- | --- | --- | --- | --- | --- | --- | --- | --- | --- | --- | --- | --- | --- | --- | --- | --- | --- | --- | --- | --- | --- | --- | --- | --- | --- | --- | --- | --- | --- | --- | --- | --- | --- | --- | --- | --- | --- | --- | --- | --- | --- | --- | --- | --- | --- | --- | --- | --- | --- | --- | --- | --- | --- | --- | --- | --- | --- | --- | --- | --- | --- | --- | --- | --- | --- | --- | --- | --- | --- | --- | --- | --- | --- | --- | --- | --- | --- | --- | --- | --- | --- | --- | --- | --- | --- | --- | --- | --- | --- | --- | --- | --- | --- | --- | --- | --- | --- | --- | --- | --- | --- | --- | --- | --- | --- | --- | --- | --- | --- | --- | --- | --- | --- | --- | --- | --- | --- | --- | --- | --- | --- | --- | --- | --- | --- | --- | --- | --- | --- | --- | --- | --- | --- | --- | --- | --- | --- | --- | --- | --- | --- | --- | --- | --- | --- | --- | --- | --- | --- | --- | --- | --- | --- | --- | --- | --- | --- | --- | --- | --- | --- | --- | --- | --- | --- | --- | --- | --- | --- | --- | --- | --- | --- | --- | --- | --- | --- | --- | --- | --- | --- | --- | --- | --- | --- | --- | --- | --- | --- | --- | --- | --- | --- | --- | --- | --- | --- | --- | --- | --- | --- | --- | --- | --- | --- | --- | --- | --- | --- |
|  |  |  |  |  |  |
|  |  |  |  |  |  |
|  |  |  |  |  |  |
|  |  |  |  |  |  |
